# Bio-Inspired Phase-Aware Skill Graph for Robust Long-Horizon Robotic Manipulation with Promptable Control

**DOI:** 10.3390/biomimetics11090622

**Published:** 2026-09-02

**Authors:** Jincheng Sun, Yang Luo, Yaqi Chu, Xiao Wang, Yunxiang Jiang, Xingang Zhao, Yiwen Zhao

**Affiliations:** 1 College of Information Science and Engineering, Northeastern University, Shenyang 110819, China; sunjc3@mails.neu.edu.cn; 2 Shenyang Institute of Automation, Chinese Academy of Sciences, Shenyang 110016, China; chuyaqi@sia.cn (Y.C.); wangxiao1@sia.cn (X.W.); jiangyunxiang@sia.cn (Y.J.); zhaoxingang@sia.cn (X.Z.); 3 University of Chinese Academy of Sciences, Beijing 101408, China

**Keywords:** biomimetic manipulation, motor primitives, phase-aware control, long-horizon manipulation, multimodal perception, skill graph, promptable control, failure recovery

## Abstract

Long-horizon robotic manipulation requires coordinating multiple motor primitives under uncertainty, especially in contact-rich and changing environments. Existing end-to-end visuomotor policies often lack explicit temporal structure, causing brittle execution and limited recovery after disturbances. Inspired by biological motor control, where reusable primitives are organized through phase decomposition and feedback-dependent transitions, we propose a bio-inspired phase-aware framework that represents execution as structured transitions over perception-grounded motor phases. The framework integrates three modules: a Multimodal Phase-and-Primitive Detector that extracts semantically and physically consistent phases from visual, proprioceptive, and force–torque signals; a Multimodal Perception Skill Graph (MPSG) that encodes feasible phase transitions and supports skipping, rollback, and recovery; and Promptable Phase Control, which converts language instructions into graph-level ordering constraints for task reordering without retraining low-level policies. Experiments on four multi-stage tasks show improved robustness, increasing the average disturbed-condition success rate from 25.0% to 73.8% relative to the monolithic Action Chunking with Transformers (ACT) baseline.

## 1. Introduction

Biological motor control systems demonstrate remarkable robustness and adaptability when performing complex manipulation and locomotion tasks in uncertain environments. Rather than relying on monolithic execution strategies, biological systems organize behavior into hierarchically structured motor primitives such as reaching, grasping, lifting, and releasing. These primitives are coordinated through phase transitions, enabling flexible adaptation to perturbations and changes in environmental conditions. Neurophysiological studies suggest that such structured control is distributed across cortical and subcortical circuits, where phase-level organization plays a central role in stabilizing motor execution and reducing error propagation across time [1,2,3,4,5,6,7,8].

In contrast, contemporary robotic manipulation systems, particularly end-to-end visuomotor policies [9,10,11,12] and large vision–language–action models [13,14,15], typically lack explicit phase-level representations. Although these methods have achieved strong performance in short-horizon tasks, they often entangle perception, planning, and control within a single policy representation, making them vulnerable to compounding errors in long-horizon execution. As a result, small perturbations in contact-rich environments can propagate through the entire trajectory, leading to cascading failures and limited recovery capability.

To address these limitations, hierarchical imitation learning and skill-based decomposition methods have been proposed [16,17,18,19,20,21,22,23,24]. While these approaches improve compositionality, they generally rely on predefined skill boundaries or fixed execution graphs determined offline. Consequently, they cannot adapt the execution structure during runtime in response to unexpected physical feedback or environmental changes.

Existing approaches primarily treat temporal structure either as a latent variable or as a fixed planning artifact. However, in real-world manipulation, task feasibility is not static; it depends on evolving physical interactions such as contact states, force transitions, and geometric uncertainty [25,26,27,28]. This reveals a fundamental gap: current systems lack a perception-grounded, phase-level execution representation that supports dynamic reconfiguration during execution. This observation motivates a shift in perspective: robotic manipulation should be modeled as a phase-organized motor system with adaptive transitions between reusable primitives, rather than a fixed trajectory execution problem.

The term “bio-inspired” in this work refers to a functional abstraction of biological motor organization rather than a direct reproduction of neural circuitry. Specifically, the proposed framework adopts four principles: hierarchical separation between intention and motor execution, composition through reusable primitives, sensory-feedback-dependent phase transitions, and localized correction following disturbances. These principles directly motivate the separation between PPC and low-level policies, the node-based organization of motor primitives, multimodal perceptual gating, and rollback-based recovery, respectively.

In this work, we propose a biomimetic phase-aware manipulation framework that explicitly incorporates phase structure into long-horizon robotic execution.

First, a Multimodal Phase-and-Primitive Detector (PPD) segments demonstrations into semantically meaningful motor phases by leveraging visual, proprioceptive, and force–torque modalities, enabling physically grounded boundary detection under uncertainty.

Second, MPSG organizes these primitives into a structured execution topology, where nodes represent perception-conditioned motor skills and edges encode feasible transitions. Unlike static skill graphs, MPSG supports runtime reconfiguration, enabling phase skipping, rollback, and recovery based on real-time perception.

Third, Promptable Phase Control (PPC) provides a language-to-graph interface for execution-time task-order reconfiguration. PPC converts language instructions into deterministic graph-level constraints that reorder or activate previously learned primitives without retraining their low-level policies. The scope of this capability is limited to new execution orders composed from the existing primitive library and validated graph nodes; it does not imply zero-shot acquisition of unseen skills, objects, or environments.

## 2. Related Work

### 2.1. Biological Motor Control and Hierarchical Action Structure

Biological motor systems provide a foundational inspiration for robotic manipulation due to their ability to achieve robust and adaptive behavior under uncertainty. In particular, human motor control is organized in a hierarchical manner, where complex behaviors are decomposed into reusable motor primitives such as reaching, grasping, manipulation, and release [1,2,3,4,5,6,7,24]. These primitives are coordinated through phase transitions that enable flexible adaptation to environmental perturbations and partial observability [3,4,5,6,7,8].

Neuroscientific studies further suggest that motor execution is not purely continuous, but is instead structured around discrete phase-level representations distributed across cortical and subcortical circuits [7,8]. Such organization allows biological systems to maintain stability while dynamically switching between behavioral modes under changing task constraints. This biological principle motivates the design of phase-aware robotic systems that explicitly model temporal structure in manipulation tasks.

### 2.2. Skill Decomposition in Robotic Manipulation

Inspired by biological motor hierarchies, robotic manipulation research has explored skill decomposition and hierarchical learning frameworks. Imitation learning methods have demonstrated strong performance in short-horizon manipulation tasks by learning visuomotor policies directly from demonstrations [9,10,11,12,16,17]. However, these approaches often suffer from compounding errors and lack explicit temporal structure when extended to long-horizon tasks [16,17,21,22].

To address these limitations, hierarchical imitation learning and skill-conditioned policies have been proposed, where high-level policies select from predefined skill sets while low-level controllers execute motor commands [18,19,20,21,22,23,24]. Although these methods improve compositional generalization, they typically rely on offline-defined skill boundaries and static execution graphs, limiting their adaptability in dynamic environments.

Recent works further incorporate multimodal signals such as vision, proprioception, and force–torque feedback to improve skill segmentation and robustness in contact-rich manipulation [25,26,27,28]. Nevertheless, most existing approaches still assume fixed execution structures, preventing runtime reconfiguration under unexpected disturbances.

### 2.3. Graph-Based and Language-Conditioned Control

Graph-based representations have been widely used to model long-horizon robotic manipulation tasks. Graph-like and skill-chaining representations encode long-horizon task structure as reusable nodes and transition relations, enabling compositional reasoning and structured planning [22,23]. Generative skill chaining and related frameworks further extend this paradigm by constructing reusable behavioral representations for multi-stage manipulation tasks [25].

However, most graph-based methods operate under a planning-time assumption, where the graph structure is fixed prior to execution and remains unchanged during deployment. This limits their ability to respond to environmental changes such as contact uncertainty, execution failure, or dynamic task reordering.

In parallel, language-conditioned robotic systems have demonstrated strong capabilities in mapping natural language instructions to manipulation policies [29,30]. However, language is typically treated as a soft conditioning signal rather than a structural constraint on execution. As a result, these methods lack explicit mechanisms for modifying execution topology or recovering from phase-level failures during runtime.

This reveals a key limitation in current approaches: the absence of a perception-grounded, phase-level execution representation that jointly supports adaptation, recovery, and structural reconfiguration in long-horizon manipulation tasks [22,23,29,30].

To clarify the methodological positioning of this work, we emphasize that the proposed contribution is not a new visual backbone, temporal segmentation backbone, or low-level skill policy in isolation. Instead, the novelty lies in the system-level formulation that (i) extracts physically grounded phase abstractions from multimodal demonstrations, (ii) organizes these abstractions into a perception-conditioned execution graph with skipping, rollback, and recovery, and (iii) exposes the execution topology to language through deterministic graph-level constraints rather than soft policy conditioning. Table 1 summarizes the differences between the proposed framework and representative related approaches.

## 3. Method

### 3.1. Biological Principles and Computational Abstraction

**Figure 1:**
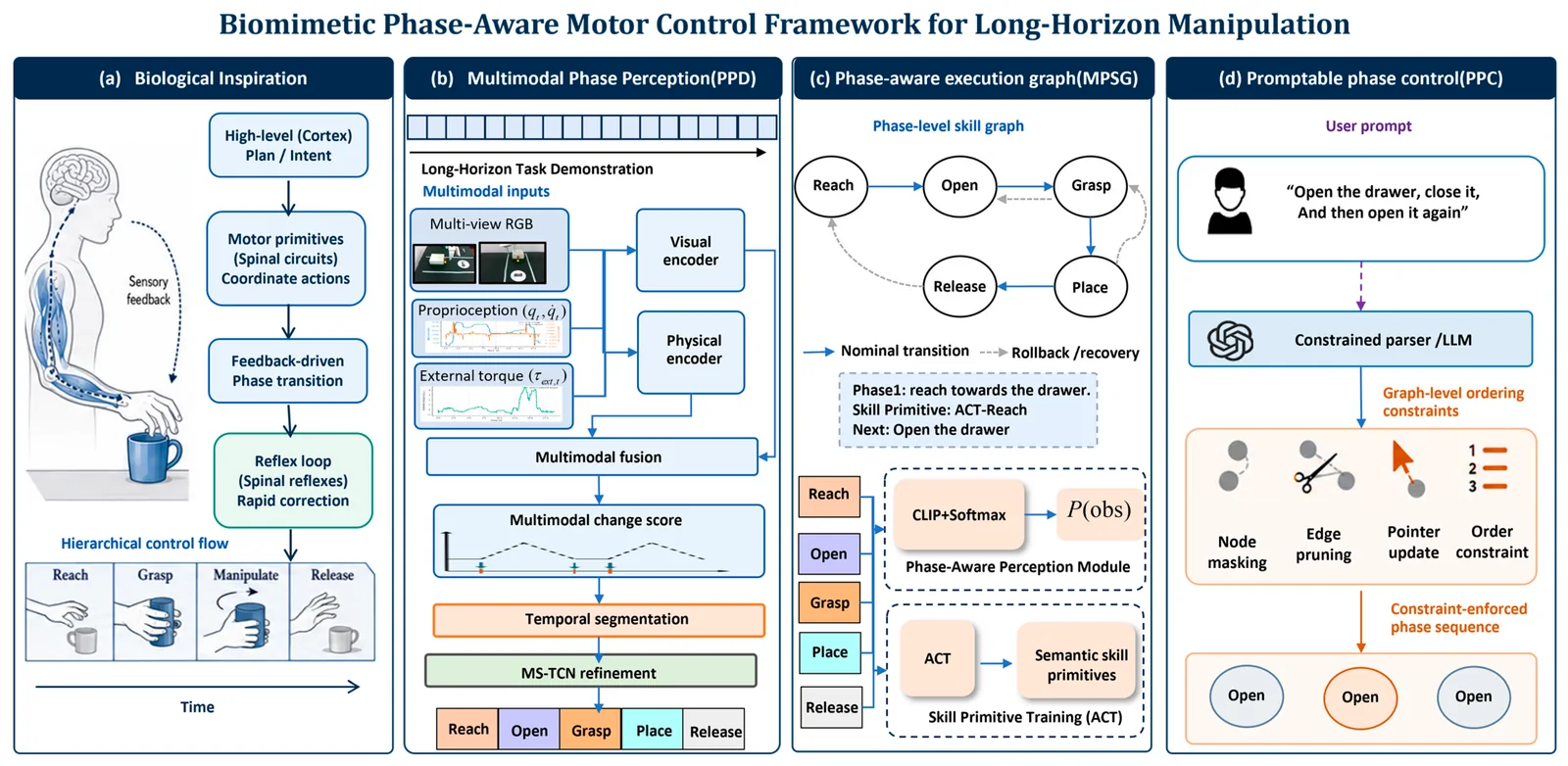
Bio-inspired phase-aware framework and its functional mapping to biological motor-control principles. The framework does not reproduce detailed neural circuitry; instead, it abstracts hierarchical intention, reusable motor primitives, sensory-feedback-dependent phase transitions, and localized corrective responses into PPC, node-level skill policies, multimodal phase perception, and graph-based recovery, respectively.

The proposed framework does not attempt to reproduce the detailed neural circuits, muscle synergies, or physiological dynamics of biological motor control. Instead, it computationally abstracts four functional principles that are consistently observed in biological motor organization: hierarchical control, reusable motor primitives, sensory-feedback-dependent phase transitions, and localized corrective responses. Details are shown in Figure 1, and the mapping between biological principles and computational abstractions is summarized in Table 2.

First, biological motor behavior is hierarchically organized, with high-level intention and task organization separated from lower-level motor execution. This principle motivates the separation between Promptable Phase Control (PPC), the phase-level execution graph, and the node-specific visuomotor policies. PPC modifies task-level constraints, whereas the underlying ACT policies remain responsible for local motor execution.

Second, complex behavior is composed from reusable motor primitives rather than generated as a single monolithic trajectory. In the proposed framework, each MPSG node encapsulates a locally executable motor primitive together with its multimodal perceptual anchor. The same primitive can therefore be reused across different execution sequences without retraining its low-level policy.

Third, biological phase transitions depend on sensory evidence rather than on elapsed time alone. This principle is abstracted by the Multimodal Phase-and-Primitive Detector (PPD) and the perception-gated graph traversal mechanism. Visual, proprioceptive, and force–torque observations jointly determine phase boundaries and phase feasibility, thereby preventing purely time-driven progression.

Fourth, biological motor systems tend to contain disturbances through local corrective responses instead of restarting an entire behavior. MPSG abstracts this principle through skipping, rollback, and recovery transitions. These graph operations do not model physiological reflexes directly; rather, they provide a functional analogue of localized feedback-based correction at the phase level.

### 3.2. Multimodal Phase-and-Primitive Detector (PPD)

To clarify the task decomposition, we distinguish a phase from a primitive. A phase denotes a temporally contiguous interval characterized by locally consistent sensory–motor interaction patterns, whereas a primitive denotes the semantic action category assigned to that interval, such as Reach, Grasp, Move, Place, or Release. In other words, the phase specifies when a consistent interaction mode occurs, while the primitive specifies what action that segment represents.

To improve downstream execution robustness, particularly in contact-rich manipulation tasks, the extracted segments are rigorously required to satisfy dual criteria: semantic and physical consistency. Semantic consistency requires that frames within a phase share the same dominant primitive semantics, such as Reach, Grasp, Move, Place, or Release. Physical consistency requires that proprioceptive and force–torque patterns remain locally coherent within the segment, while phase boundaries are aligned with salient interaction changes, such as contact onset, gripper closure, or force release, rather than superficial visual fluctuations.

**Figure 2:**
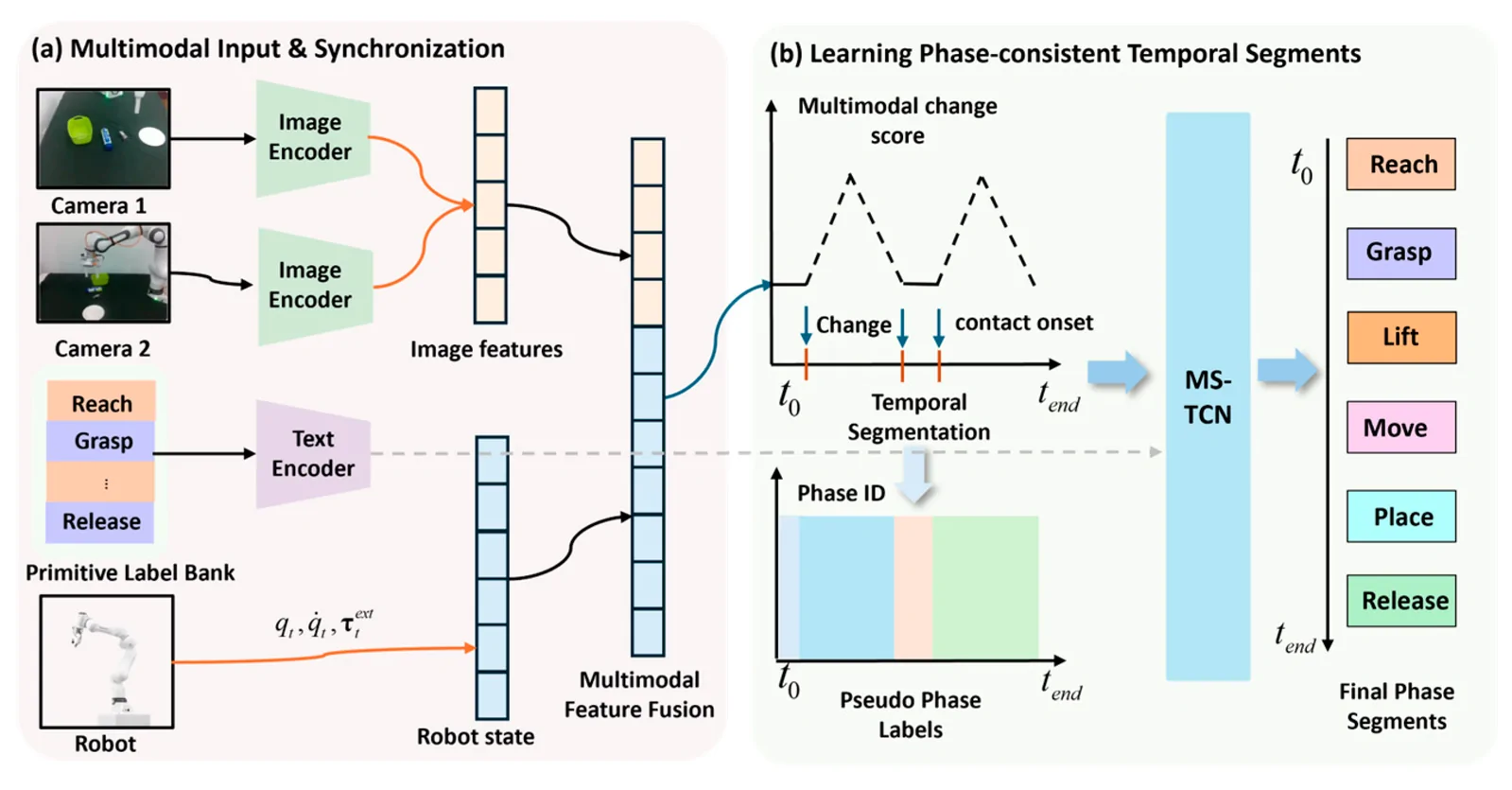
Overview of the Multimodal Phase-and-Primitive Detector (PPD). (**a**) Multi-view images and the complete robot observation are synchronized at 30 Hz. For phase segmentation, the physical branch uses a compact five-dimensional contact-oriented representation derived from the binary gripper state and external joint torques, while joint positions and velocities remain available to the robot-control and node-level policy components. The two visual embeddings and the encoded physical feature are fused before temporal modeling. (**b**) Candidate phase boundaries are generated from the fused features and refined by MS-TCN to produce temporally consistent semantic phase segments. Ellipses in the state sequence indicate omitted intermediate manipulation steps that are not the focus of this figure.

Driven by these criteria, long-horizon demonstrations are decomposed by the Multimodal Phase-and-Primitive Detector (PPD) into a structured sequence of semantically and physically consistent phase segments rather than unstructured frame-level labels, as illustrated in Figure 2. Each observation consists of multi-view RGB images, robot proprioceptive states, and force–torque signals. Language prompts are not used to determine temporal boundaries. Instead, they are used only after boundary detection to assign semantic primitive names to the segmented phases. Visual features are extracted via a pre-trained CLIP encoder [31], while a compact five-dimensional physical descriptor derived from the binary gripper state and external joint-torque signals is encoded using a lightweight MLP. Joint positions and velocities remain part of the complete robot observation and node-level policy input but are not directly concatenated into the reported PPD physical branch. These modality-specific features are fused into a unified representation for change-point detection. Phase boundaries are identified by detecting multimodal consistency shifts in the fused trajectory, ensuring alignment with physical interaction events rather than visual noise. The resulting segments are refined using an MS-TCN [32,33] to produce temporally consistent semantic primitives.

Multimodal Observation Representation: At each time step t, the robot observes a set of synchronized multimodal signals:

(1) $$O_{t}=\left\{{\left\{I_{t}^{\left(r\right)}\right\}}_{r=1}^{N_{v}},q_{t},{\dot{q}}_{t},\tau_{t}^{\mathrm{ext}},g_{t}\right\},$$

where $I_{t}^{\left(r\right)}\in {\mathbb{R}}^{H\times W\times 3}$ is the RGB image from the r-th camera, $N_{v}$ is the number of camera views, $q_{t}\in {\mathbb{R}}^{n_{q}}$ and ${\dot{q}}_{t}\in {\mathbb{R}}^{n_{q}}$ denote the robot joint positions and velocities, respectively, and $g_{t}$ denotes the gripper state. $\tau_{t}^{\mathrm{ext}}\in {\mathbb{R}}^{n_{q}}$ denotes the estimated external joint torque. Equation (1) describes the complete observation available to the robotic system. Joint positions and velocities are used by the robot-control interface and node-level visuomotor policies; the reported phase-segmentation branch uses the compact contact-oriented representation defined below rather than directly concatenating the complete raw proprioceptive vector.

$$\begin{array}{l} p_{t}={\left[g_{t},m_{t},\Delta m_{t},a_{t},c_{t}\right]}^{⊤}\in {\mathbb{R}}^{5},\\ m_{t}={\left\|\tau_{t}^{S}\right\|}_{2},\;\Delta m_{t}=\left|{\left\|\tau_{t}^{S}\right\|}_{2}-{\left\|\tau_{t-1}^{S}\right\|}_{2}\right|,\\ u_{t}=\frac{\tau_{t}^{S}}{{\left\|\tau_{t}^{S}\right\|}_{2}+\epsilon },\;a_{t}=1-u_{t}^{⊤}u_{t-1},\\ c_{t}={\left\|\frac{1}{\left|W_{t}\right|}\sum_{j\in W_{t}}u_{j}\right\|}_{2},\;\epsilon =10^{-6},\;|W_{t}|=4. \end{array}$$

Here, $g_{t}$ is the binary gripper state, $\tau_{t}^{S}$ contains the selected contact-sensitive external-torque channels, $m_{t}$ is the torque magnitude, $\Delta m_{t}$ is the frame-to-frame torque-magnitude change, $a_{t}$ is the torque-direction change, and $c_{t}$ is the local torque-direction consistency computed over a four-frame window. Therefore, the physical representation consists of one gripper-state feature and four torque-derived contact features.

Semantic-Visual and Physical Encoding: Each camera image is encoded using OpenCLIP ViT-B/32 with the laion2b_s34b_b79k pretrained weights. The resulting image embedding is $L_{2}$-normalized:

(2) $$z_{t}^{v,r}=\frac{f_{\mathrm{CLIP}}\left(I_{t}^{\left(r\right)}\right)}{{\left\|f_{\mathrm{CLIP}}\left(I_{t}^{\left(r\right)}\right)\right\|}_{2}+\epsilon }\in {\mathbb{R}}^{512}.$$

The embeddings from the head and right cameras are concatenated:

(3) $$z_{t}^{v}=\left[z_{t}^{v,h};z_{t}^{v,r}\right]\in {\mathbb{R}}^{1024}.$$

Before physical encoding, the five channels of $p_{t}$ are standardized using channel-wise means and standard deviations computed exclusively from the training set. The standardized physical vector is processed by a two-layer physical encoder:

(4) $$z_{t}^{p}=E_{p}\left(p_{t}\right)\in {\mathbb{R}}^{64},$$

where $E_{p}$ consists of

$$\begin{array}{l} E_{p}:\mathrm{Linear}(5,32)\to \mathrm{ReLU}\to \mathrm{LayerNorm}(32)\to \mathrm{Dropout}(0.1)\to \\ \mathrm{Linear}(32,64)\to \mathrm{ReLU}\to \mathrm{LayerNorm}(64)\to \mathrm{Dropout}(0.1) \end{array}$$

Multimodal Feature Fusion: The modality-specific features are computed as

(5) $$h_{t}=E_{f}\left(\left[z_{t}^{v};z_{t}^{p}\right]\right)\in {\mathbb{R}}^{256},$$

where the concatenated feature has 1024 + 64 = 1088 dimensions. The fusion network $E_{f}$ is implemented as

Linear(1088,512)→GELU→Dropout(0.2)→Linear(512,256)→GELU→Dropout(0.1).

The resulting 256-dimensional fused feature sequence ${\left\{h_{t}\right\}}_{t=1}^{T}$ is used for change-point detection and subsequently provided to the MS-TCN.

Physically Grounded Segmentation: Candidate phase boundaries are generated from the fused feature sequence ${\left\{h_{t}\right\}}_{t=1}^{T}$ using binary segmentation with a squared-Euclidean segment cost. For a temporal interval [a,b], the segment cost is

(6) $$C(a,b)=\sum_{t=a}^{b}{\left\|h_{t}-{\bar{h}}_{a:b}\right\|}_{2}^{2},\;{\bar{h}}_{a:b}=\frac{1}{b-a+1}\sum_{t=a}^{b}h_{t}.$$

In the reported implementation, binary segmentation uses a penalty of 2.0, a minimum segment size of 3 frames, and a jump interval of 5 frames. The resulting pseudo-phase labels are temporally smoothed using an 11-frame window. During post-processing, a minimum phase duration of 15 frames (0.5 s at 30 Hz) is enforced before the pseudo-label sequence is used to supervise the MS-TCN.

Temporal Phase-and-Primitive Modeling: The candidate boundaries obtained from change-point detection are further refined using a Multi-Stage Temporal Convolutional Network (MS-TCN) to enforce global temporal consistency and eliminate boundary jitter. Let $C_{p}$ denote the number of primitive classes, $p_{t}={\left[p_{t,1},\ldots ,p_{t,C_{p}}\right]}^{⊤}$ denote the MS-TCN softmax output, and $y_{t}\in {\left\{0,1\right\}}^{C_{p}}$ denote the one-hot training label.

During training, the MS-TCN is supervised using frame-wise pseudo-labels generated by the physically grounded change-point detector. For each task, 20 development demonstrations were partitioned at the episode level into 16 training episodes and 4 validation episodes before temporal-window extraction. Across the four tasks, this resulted in 64 training episodes and 16 validation episodes. The training partition was used for gradient-based optimization, whereas the validation partition was used for early stopping, checkpoint selection, and hyperparameter selection.

An additional set of 20 successful demonstrations per task was independently collected and manually annotated, resulting in an 80-episode manual test benchmark. This benchmark was disjoint from the training and validation partitions and was used exclusively for the final quantitative evaluation of phase-segmentation performance after all model configurations had been fixed. The reported MS-TCN is optimized using frame-wise cross-entropy loss:

(7) $$L_{seg}=-\frac{1}{T}\sum_{t=1}^{T}\sum_{c=1}^{C_{p}}y_{t,c}\log(p_{t,c}+\epsilon ).$$

Following the inference of frame-wise probabilities, continuous frames sharing identical maximum probability labels are aggregated into a structured set of discrete phase segments:

(8) $$S={\left\{s_{m}\right\}}_{m=1}^{M},\;s_{m}=(l_{m},t_{m}^{s},t_{m}^{e},\phi_{m})$$

where M denotes the total number of partitioned phases within a single demonstration trajectory. For the m-th phase segment $s_{m}$, $l_{m}$ represents the assigned primitive label, $t_{m}^{s}$ and $t_{m}^{e}$ signify the starting and ending time frames respectively, and $\phi_{m}$ constitutes the multimodal perceptual anchor extracted within the temporal extent $\left[t_{m}^{s},t_{m}^{e}\right]$. This segment-level formulation provides the necessary structural primitives and sensory anchors to directly initialize the downstream MPSG.

The detailed architecture, preprocessing procedure, and training configuration of PPD are provided in Appendix A, Table A1. All configurations were fixed before final evaluation and kept unchanged across the four evaluated tasks.

### 3.3. Multimodal Perception Skill Graph (MPSG)

While PPD decomposes demonstrations into semantically labeled and physically grounded phase segments, robust long-horizon execution requires an additional structure to model how these phases can be selected, skipped, repeated, or recovered during runtime. A fixed sequential execution order is insufficient in disturbed environments, because the physical preconditions of a phase may become invalid, already satisfied, or temporarily ambiguous. To address this problem, we introduce the MPSG, a perception-conditioned execution topology that represents manipulation as graph traversal over reusable phase-level skill units.

In MPSG, each node corresponds to a locally executable skill primitive equipped with a multimodal perceptual anchor, and each directed edge represents a feasible transition observed in demonstrations or validated through successful recovery traces. Therefore, the graph does not merely encode the nominal task order; it also stores alternative transitions that support runtime branching, phase skipping, rollback, and failure recovery. This formulation allows long-horizon manipulation to be executed as perception-grounded phase navigation rather than as a rigid temporal sequence.

Incremental Graph Construction: Unlike rigid state machines with manually predefined connections, the topology of MPSG is built dynamically. The initial directed graph $G=(V,E),V={\left\{\nu_{i}\right\}}_{i=1}^{N},E\subseteq V\times V.$ is constructed by aggregating the PPD-segmented trajectories.

Nodes (V): Each node $\nu_{i}=\left(l_{i},\pi_{i},\phi_{i}\right)\in V$, where $l_{i}\in L$ is the semantic primitive label, $\pi_{i}$ is the node-specific ACT policy, and $\phi_{i}$ is the multimodal perceptual anchor associated with the physical context of that node. The symbols i and j index graph-node instances, whereas t denotes time and k is reserved for indexing phase segments. Semantic names such as Open, Place, and Close are elements of the primitive-label set L rather than node indices. Multiple nodes may share the same semantic label, $l_{i}=l_{j}$, while retaining different perceptual anchors or execution contexts. Conversely, segments with sufficiently similar semantic and multimodal contexts may be merged into the same graph node.

Edges (E): Directed edges $e_{ij}=\left(\nu_{i},\nu_{j},n_{ij},r_{ij}\right)\in E,$ represent feasible transitions explicitly observed and logged by the system. $r_{ij}\in \{nominal,skip,rollback,recovery\}$, $n_{ij}$ denotes the total number of observed transitions from phase i to phase j across both the expert demonstrations and the validated recovery traces.

Experience-driven recovery behaviors are explicitly encoded in the graph through an incremental self-expansion mechanism. During graph construction and pre-evaluation development, validated recovery trajectories may be incorporated as recovery edges. For all quantitative experiments reported in this paper, graph construction is completed before evaluation and the resulting graph is frozen. No node, edge, edge count, perceptual anchor, or node-level policy is modified during evaluation.

Phase-Aware Graph Traversal: During execution, MPSG acts as a probabilistic state estimator and navigator. At each step t, the system determines the next skill node by evaluating the Perceptual Gating Score:

(9) $$s_{j}(t)=\omega_{v}\cos\left(z_{t}^{v},\phi_{j}^{v}\right)+\omega_{p}\cos\left(z_{t}^{p},\phi_{j}^{p}\right),$$

where $\omega_{v},\omega_{p}\geq 0,\omega_{v}+\omega_{p}=1$.

Transition prior:

(10) $$P_{E}(j|i)=\frac{n_{ij}+\epsilon_{E}}{\sum_{m\in N^{+}(i)}\left(n_{im}+\epsilon_{E}\right)},$$

where $N^{+}(i)$ denotes the outgoing neighbors of node $\nu_{i}$ and $\epsilon_{E}>0$ is a smoothing constant.

Equation (10) represents a static prior obtained from demonstrations and validated recovery traces. At execution time t, this prior is reweighted using the observation-dependent perceptual score $s_{j}(t)$ of each candidate node:

(11) $${\bar{P}}_{t}(j|i_{t})=\frac{\exp\left(s_{j}(t)/\tau_{g}\right)P_{E}{\left(j|i_{t}\right)}^{\lambda_{E}}}{\sum_{m\in N^{+}(i_{t})}\exp\left(s_{m}(t)/\tau_{g}\right)P_{E}{\left(m|i_{t}\right)}^{\lambda_{E}}},$$

where $\tau_{g}>0$ is the softmax temperature controlling the sharpness of the normalized candidate scores; $\lambda_{E}\geq 0$ denotes the weight assigned to the empirical transition prior; and $i_{t}$ represents the index of the currently active graph node at time step t.

This mechanism enables dynamic execution:

Automatic Skipping: Suppose that the currently active node $v_{i}$ has the primitive label $l_{i}=Open$, while a candidate successor $v_{j}$ represents a post-opening phase, such as $l_{j}=Place$. If the drawer has already been opened externally, the current observation becomes more consistent with the perceptual anchor $\phi_{j}$ than with $\phi_{i}$. Consequently, $s_{j}(t)$ increases and the graph pointer may advance to the context-consistent successor, provided that the graph-connectivity and feasibility conditions are satisfied.

Robust Transitioning: Transitions are not blindly triggered by time; they are gated by physical reality (e.g., verifying a torque spike before transitioning from Grasp to Lift), ensuring that the robot never proceeds to the next stage prematurely.

Constraint Injection: Each parsed action $a_{i}$ is deterministically mapped to a graph operator: Node masking: $(v_{i})\Rightarrow \alpha_{i}=0$; Pointer update: $(v_{j})\Rightarrow v_{t}\leftarrow v_{j}$; Edge rewiring: $(v_{i},v_{j})\Rightarrow e_{ij}\in E$.

Constraint-Aware Graph Execution: Let $v_{i}$ denote the currently active node, $E_{t}$ the active edge set, and $M_{t}$ the node-mask set at time t. In the absence of prompt-driven constraints, $E_{t}=E$ and $M_{t}=\emptyset$. Based on the perceptual score $s_{j}(t)$ and the normalized transition confidence $P_{t}(j|i)$ defined above, the admissible candidate set is constructed as

(12) $$A_{t}=\left\{j|(\nu_{i_{t}},\nu_{j})\in E_{t},\;j\notin M_{t},\;s_{j}(t)\geq \eta_{\phi }\right\},$$

where $\eta_{\phi }$ is the perceptual-feasibility threshold.

(13) $$P_{t}(j|i_{t},M_{t},E_{t})=\frac{I[j\in A_{t}]{\bar{P}}_{t}(j|i_{t})}{\sum_{m}I[m\in A_{t}]{\bar{P}}_{t}(m|i_{t})}$$

The next active node is selected via

(14) $$i_{t+1}=\underset{j\in A_{t}}{\mathrm{argmax}}\;P_{t}(j|i_{t},M_{t},E_{t})$$

Threshold Application: Four fixed thresholds are used in graph traversal. A candidate node first satisfies the perceptual-feasibility condition $s_{j}(t)\geq \eta_{p}$, with $\eta_{p}=0.70$. After combining the perceptual score with the empirical transition prior, node activation additionally requires $P_{t}(j|i)\geq \eta_{t}$, where $\eta_{t}=0.80$. A nominal forward transition is enabled only after the current phase satisfies its completion criterion with confidence at least $\eta_{c}=0.85$. Nonadjacent forward skipping uses the stricter condition $s_{j}(t)\geq \eta_{s}=0.90$. All thresholds are fixed before evaluation and are identical across trials.

To guarantee robust execution and resolve edge cases during long-horizon manipulation, the graph transition logic enforces the following deterministic safeguards:

Tie-Breaking: If multiple nodes yield identical maximum probabilities, tie-breaking prioritizes the node with the highest empirical occurrence $n_{i_{t}j}$ to favor historically reliable transitions.

Admissible Set Depletion ($A_{t}=\emptyset$): If no valid transitions exist, the framework triggers a localized rollback to the most recent stable phase. or when no feasible nominal or skipping successor remains. A stable rollback target is defined as a previously executed node that: (1) was successfully completed earlier in the current episode; (2) is connected to the current node by a validated rollback or recovery edge; and (3) satisfies the current perceptual-feasibility condition $s_{j}(t)\geq \eta_{p}$. If multiple rollback targets satisfy these conditions, the most recently completed feasible node is selected. The graph pointer is reassigned to this node, and its associated node-level policy is reactivated.

Recovery Loop Prevention: Because rollback and recovery edges may introduce cycles, the executor maintains a trial-level recovery counter $N_{rec}^{\max}$. A recovery attempt is counted only when a rollback or recovery edge is actually activated. Multiple control cycles spent executing the same recovery node do not constitute additional attempts. The counter is initialized to zero at the beginning of every trial and incremented once per activated rollback/recovery transition. Once $n_{rec}=N_{rec}^{\max}=3$. Further recovery transitions are disabled when $n_{rec}>N_{rec}^{\max}$. If no feasible forward candidate is available at that point, the system performs a safe stop and records a recovery failure. In all experiments, $N_{rec}^{\max}=3$, as summarized in Appendix A, Table A3.

Feasibility-Gated Reconfiguration: Graph-edit directives, including FORCE_JUMP, remain subordinate to the perception-grounded execution constraints of MPSG. A requested target node is activated only when it exists in the current graph, is reachable under the active graph constraints, and satisfies the perceptual-feasibility condition $s_{j}(t)\geq \eta_{p}$. If any of these conditions is violated, the command is rejected, the current graph pointer and active graph constraints remain unchanged, and the low-level policy associated with the infeasible target is not executed. This mechanism prevents language-derived graph commands from overriding the physical admissibility conditions of the robot execution process.

The numerical thresholds and deterministic execution parameters used by MPSG are summarized in Appendix A, Table A3. All values were fixed before the final evaluation and kept unchanged across tasks and baselines.

### 3.4. Promptable Phase Control (PPC)

To expose the MPSG as a controllable phase-level topology, Promptable Phase Control (PPC) is introduced. Rather than functioning as a conventional task planner that autoregressively generates action sequences, PPC is strictly formulated as a language-to-graph-constraint compiler coupled with a feasibility-aware graph edit interface. Unlike language-conditioned visuomotor policies that treat natural language as a soft, latent conditioning signal for end-to-end control, PPC translates language into deterministic structural constraints applied directly to the execution graph. The main notation used throughout this paper is summarized in Table 3.

Semantic-to-Topological Projection: Let p∈P denote a natural-language instruction and $\Sigma_{G}$ denote the predefined graph-operation grammar. The constrained language parser is defined as

(15) $$\Phi_{\mathrm{LLM}}:(p,\Sigma_{G})\mapsto a=(a_{1},\ldots ,a_{R}),$$

where R is the number of parsed graph operations. Every operation is defined as

(16) $$a_{r}=\left(o_{r},u_{r},\sigma_{r}\right),$$

where $o_{r}\in O_{G}$ is the operator type, $u_{r}$ is an ordered tuple of node arguments, and $\sigma_{r}$ specifies the operation scope:

(17) $$O_{G}=\{Mask,Unmask,SetPointer,AddEdge,RemoveEdge,Order\}.$$

In the reported implementation, PPC is used to impose an ordered sequence over existing graph nodes. It neither creates a previously unseen node nor synthesizes a new low-level skill. The parsed sequence is converted into a temporary node mask and effective edge set, which are subsequently executed using the perception-gated MPSG traversal rule. If the requested sequence references an unavailable node, violates graph connectivity, or fails the current perceptual-feasibility check, the constraint is rejected. The current graph pointer and active graph constraints remain unchanged, and no low-level policy associated with the infeasible target is activated.

The LLM is bounded by a fixed instruction schema to emit only valid graph-edit operations (e.g., mask, set_pointer, rewire, force_jump), guaranteeing that natural language is compiled into a deterministic grammar rather than interpreted as free-form commands. These operations reconfigure the admissible phase-transition space while leaving the underlying low-level policies strictly frozen, thereby establishing a robust interface for runtime intervention. By manipulating the graph topology rather than altering policy weights, the system can enforce temporal constraints, bypass irrelevant phases, and proactively redirect execution toward feasible recovery nodes under external disturbances. Furthermore, to ensure physical stability during reconfiguration, the graph-edit interface is strictly feasibility-aware. A generated operation $a_{k}$ is accepted if and only if the target node $v_{k}$ satisfies the perceptual feasibility check governed by multimodal anchors; If the requested target node does not satisfy the perceptual-feasibility condition, the graph-edit command is rejected and the current graph pointer is retained. No low-level policy associated with the infeasible target is activated. Ultimately, this paradigm allows language to act as a hard, physically grounded topological constraint, fundamentally decoupling high-level human intervention from low-level motor generation.

Prompt-Driven Primitive-Order Reconfiguration: Because PPC modifies graph-level constraints rather than low-level policy parameters, it can impose a previously unevaluated execution order over the existing primitive library without policy retraining. For example, a nominal sequence A→B→C can be constrained to C→B→A, provided that the requested primitives and their corresponding graph nodes have already been learned and that the resulting transitions pass the perceptual-feasibility checks. This capability is restricted to recombination and reordering of previously trained primitives within the evaluated task domain. PPC does not acquire a new motor skill, infer a policy for an unseen object, construct an unobserved primitive, or guarantee transfer to a previously unseen environment.

PPC uses Kimi K2.6, accessed through an API, as a high-level semantic parser. The parser is invoked on demand once per new language instruction. The observed parsing latency is approximately 1 s and receives the user instruction together with the currently available primitive nodes in the MPSG. It converts the instruction into an ordering constraint over existing graph nodes and does not directly generate robot actions or low-level control commands. The parsed ordering constraint is subsequently processed by the MPSG executor, which searches for an executable graph route according to graph connectivity and the current perceptual-feasibility scores. Therefore, the language model specifies the intended task order, whereas the MPSG determines how that order can be physically executed.

Language-to-Graph Interface: Given the currently available primitive labels and a free-form natural-language instruction, the semantic parser returns a JSON-formatted suggested graph containing an ordered node list and corresponding directed edges. The parser is restricted to the supplied primitive vocabulary and cannot introduce unseen primitives or low-level actions. The suggested graph is not executed directly. It first undergoes deterministic syntax, schema, node-existence, and graph-validity checks. Valid outputs are converted into temporary MPSG constraints, while invalid outputs are rejected and leave the active graph unchanged. All accepted targets remain subject to the perceptual-feasibility checks of MPSG during execution.

The PPC execution flow is summarized in Algorithm 1.
**Algorithm 1** Promptable Phase-Aware Execution (PPC)**Input**: MPSG G=(V,E), node policies {*π_i_*}, perceptual anchors *{**Φ**_i_}*, optional language instruction *p* 1: Initialize the active node $v_{i}$ 2: $E_{t}\leftarrow E$, $M_{t}\leftarrow \emptyset$, $n_{rec}\leftarrow 0$ 3: **While** the task is not terminated **do** 4:        Acquire the synchronized observation $O_{t}$ 5:        **if** a new instruction p is received **then** 6:              Parse p into an ordered primitive sequence $Q=\left(l^{\left(1\right)},\ldots ,l^{\left(L\right)}\right)$ 7:              Resolve each label in Q to an existing graph node 8:              **if** all labels are resolved, the ordering is graph-consistent **then** 9:                       Construct tentative constraints $\left(M\prime_{t},E\prime_{t}\right)\leftarrow \mathrm{InjectOrder}\left(G,Q\right)$ 10:                     **if** the requested target is perceptually feasible **then** 11:                            $\;\left(M_{t},E_{t}\right)\leftarrow \left(M\prime_{t},E\prime_{t}\right)$ 12:                     **else** 13:                              Reject the instruction and retain $\left(M_{t},E_{t}\right)$ 14:                     **end if** 15:               **else** 16:                     Reject the instruction and retain the current graph 17:               **end if** 18:         **end if** 19:         Compute the perceptual scores $s_{j}\left(t\right)$ for enabled outgoing nodes 20:         Construct the admissible set $A_{t}\left(i\right)$
21:         **if** $A_{t}\left(i\right)\neq \emptyset$ **then** 22:               Select $j^{*}=\arg\max_{j\in A_{t}(i)}P_{t}(j|i,M_{t},E_{t})$ 23:               Update the active node: $i\leftarrow j^{*}$ 24:          **else if** a feasible rollback target exists $n_{rec}<N_{rec}^{max}$ $\pi_{i}\left(O_{t}\right)$ i
25:                 Set i to the most recent feasible stable node 26:                 $n_{\mathrm{rec}}\leftarrow n_{\mathrm{rec}}+1$ 27:          **else** execute a safe stop 28:                 record the trial as a recovery failure 29:                 **break** 30:          **end if** 31:          Execute the node-level policy $\pi_{i}(O_{t})$ 32: **end while**

## 4. Results

We evaluate the proposed framework on four real-world long-horizon manipulation tasks involving sequential pick-and-place, contact-rich insertion/removal, multi-object rearrangement, and ambiguous-state manipulation. The experiments are designed to verify three hypotheses: (i) sensory-feedback-dependent phase organization should produce more reliable phase boundaries than visual observation alone, particularly under occlusion and contact ambiguity; (ii) localized phase-level correction should reduce premature transitions and improve recovery compared with monolithic or fixed sequential execution; and (iii) hierarchical separation between task-level intention and low-level motor execution should enable task-order reconfiguration without retraining the underlying skill policies.

All methods are evaluated using task success rate (TSR), premature transition rate (PTR), order compliance, and phase-segmentation metrics. For all skill-based methods, the same node-level ACT policies are used. The monolithic ACT baseline is trained with the same demonstrations and comparable backbone capacity but without explicit phase decomposition or graph-level execution control.

### 4.1. Experimental Setup and Evaluation Protocol

**Figure 3:**
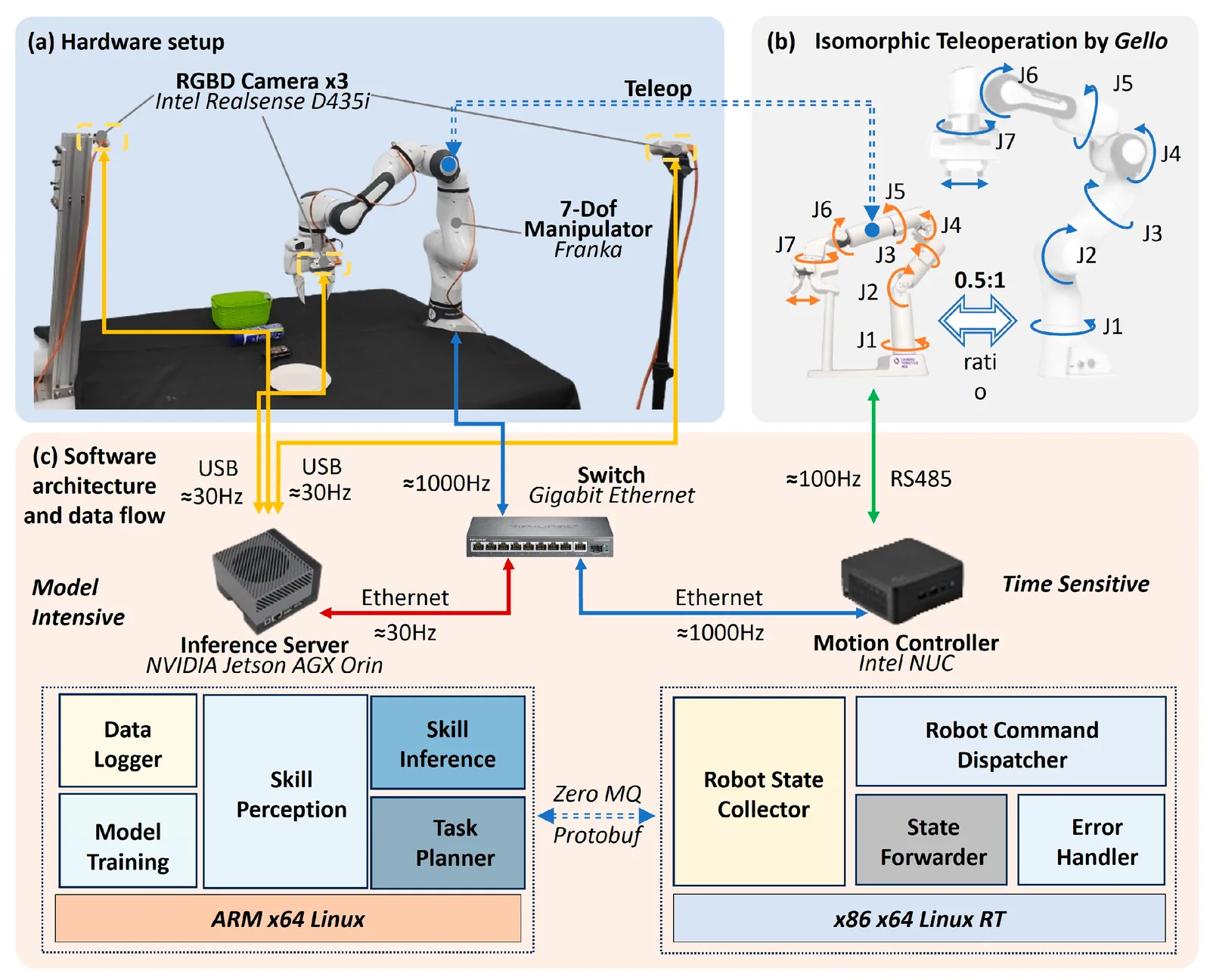
Overview of the robotic system architecture. (**a**) Experimental platform with a torque-controlled Franka Emika Panda robot and multi-view RGB-D perception. (**b**) Isomorphic teleoperation via Gello, mapping operator joint motions to robot joint commands. (**c**) Software architecture and data flow. Model inference, multimodal skill perception, and local graph execution run on an NVIDIA Jetson AGX Orin, whereas time-critical motion control is executed on an Intel NUC with real-time Linux. The optional PPC language parser is accessed through an external API.

Experimental Setup: As shown in Figure 3, the experimental platform supports real-time, multi-view perception and teleoperated skill demonstration. It integrates a 7-DoF Franka Emika Panda robotic arm, a Gello isomorphic teleoperation device, and multiple depth cameras for synchronized RGB-D sensing. Computation is distributed across an NVIDIA Jetson AGX Orin (NVIDIA Corporation, Santa Clara, CA, USA) for model inference, skill perception, and high-level reasoning, and an Intel NUC for time-critical control. The system adopts a distributed runtime architecture. Model inference, multimodal skill perception, and MPSG traversal are executed on an NVIDIA Jetson AGX Orin, whereas the time-critical robot-control loop runs on an Intel NUC under real-time Linux. The two computers communicate through Gigabit Ethernet.

The runtime system follows an asynchronous multi-rate architecture. The time-critical robot controller operates at 1 kHz on the real-time control computer, while the node-level ACT policies generate action references at 30 Hz on the Jetson AGX Orin. Online phase recognition is executed by an independent asynchronous monitoring process, with an average update latency of approximately 60 ms. The latest valid phase estimate is shared with the MPSG executor without blocking either the 30 Hz policy loop or the 1 kHz robot-control loop. Graph-pointer and constraint updates are event triggered and require approximately 10 ms after a valid phase or language constraint becomes available. Kimi K2.6 is accessed through an external API only when a new language instruction is issued. In the reported prompt-driven experiment, the language instruction was parsed before robot execution and therefore did not enter the continuous motor-control cycle. Detailed deployment modes and timing measurements are provided in Appendix B, Table A7.

For each of the four tasks, 20 demonstration episodes were manually annotated, resulting in a total of 80 annotated episodes. These episodes were excluded from model training and were used exclusively as reference annotations for evaluating the phase boundaries and primitive labels generated by the automatic phase-segmentation procedure. The annotations were produced by one of the authors. A phase boundary was assigned when a persistent change in the dominant sensory–motor interaction pattern was observed. Each resulting temporal segment was then assigned a primitive label from the predefined vocabulary. During quantitative evaluation, these manual annotations were treated as the reference labels for computing frame-wise accuracy, tolerance-based Boundary-F1, mean intersection over union, and boundary-detection metrics. Because the annotations were produced by a single annotator, inter-annotator agreement was not available. This limitation is considered when interpreting the reported segmentation results.

The task-specific completion criteria are event based rather than defined by manually specified metric thresholds. The target configuration of each primitive is determined from successful demonstrations and represented by the corresponding semantic phase and multimodal perceptual anchor. Therefore, quantities such as drawer displacement, insertion depth, block position, or knob rotation angle are not assigned additional manually tuned numerical thresholds for primitive completion. Small geometric deviations are tolerated as long as the intended semantic state is reached and the subsequent task can be completed successfully.

We evaluate four execution strategies for phase-aware execution and failure recovery:

ACT: A monolithic end-to-end visuomotor policy trained through imitation learning on complete long-horizon demonstrations.

Flat Primitive Sequence: A skill-based executor that invokes a predefined sequence of primitive policies without perception-conditioned graph traversal, phase skipping, or rollback.

SayCan-inspired Planner: A score-based high-level primitive selector that combines language compatibility and multimodal perceptual compatibility. For each candidate primitive j,

(18) $$S_{j}^{\mathrm{SC}}(t)=0.6S_{j}^{\mathrm{perc}\;}(t)+0.4S_{j}^{\mathrm{lang}\;}(p),$$

where $S_{j}^{\mathrm{perc}\;}$ uses the same multimodal perceptual scoring function as MPSG-Control and $S_{j}^{\mathrm{lang}}$ measures compatibility between the task instruction and the candidate primitive. The highest-scoring primitive is selected. The baseline uses the same frozen node-level ACT policies but maintains no explicit transition graph, rollback edges, or recovery history.

MPSG-Control: The proposed method, which performs perception-grounded phase execution using multimodal feasibility gating, graph-based transition selection, phase skipping, and rollback.

All experimental settings are summarized in Table 4. All methods are trained from the same demonstration corpus and evaluated using the same visual observations, robot states, low-level controller, hardware platform, disturbance conditions, and termination criteria. For all skill-based methods, the same node-level ACT policies and checkpoints are used. Therefore, the comparison isolates the effect of the high-level execution structure rather than differences in low-level policy capacity. The monolithic ACT baseline is trained on the original unsegmented trajectories, whereas the skill-based methods use phase-specific subsets derived from the same demonstrations. No additional task demonstrations are provided to any baseline. All methods were executed using the same robot controller, control frequency, camera streams, computing platform, disturbance protocol, and termination criteria. Evaluation trials were initialized using the same randomization ranges, and the execution order of the evaluated methods was randomized to reduce systematic bias. For all binary execution-level outcomes, we report the observed event counts and corresponding proportions. Two-sided 95% Wilson score confidence intervals are provided in Appendix D, Table A9, Table A10 and Table A11.

### 4.2. Semantic Phase Segmentation Ablation

**Figure 4:**
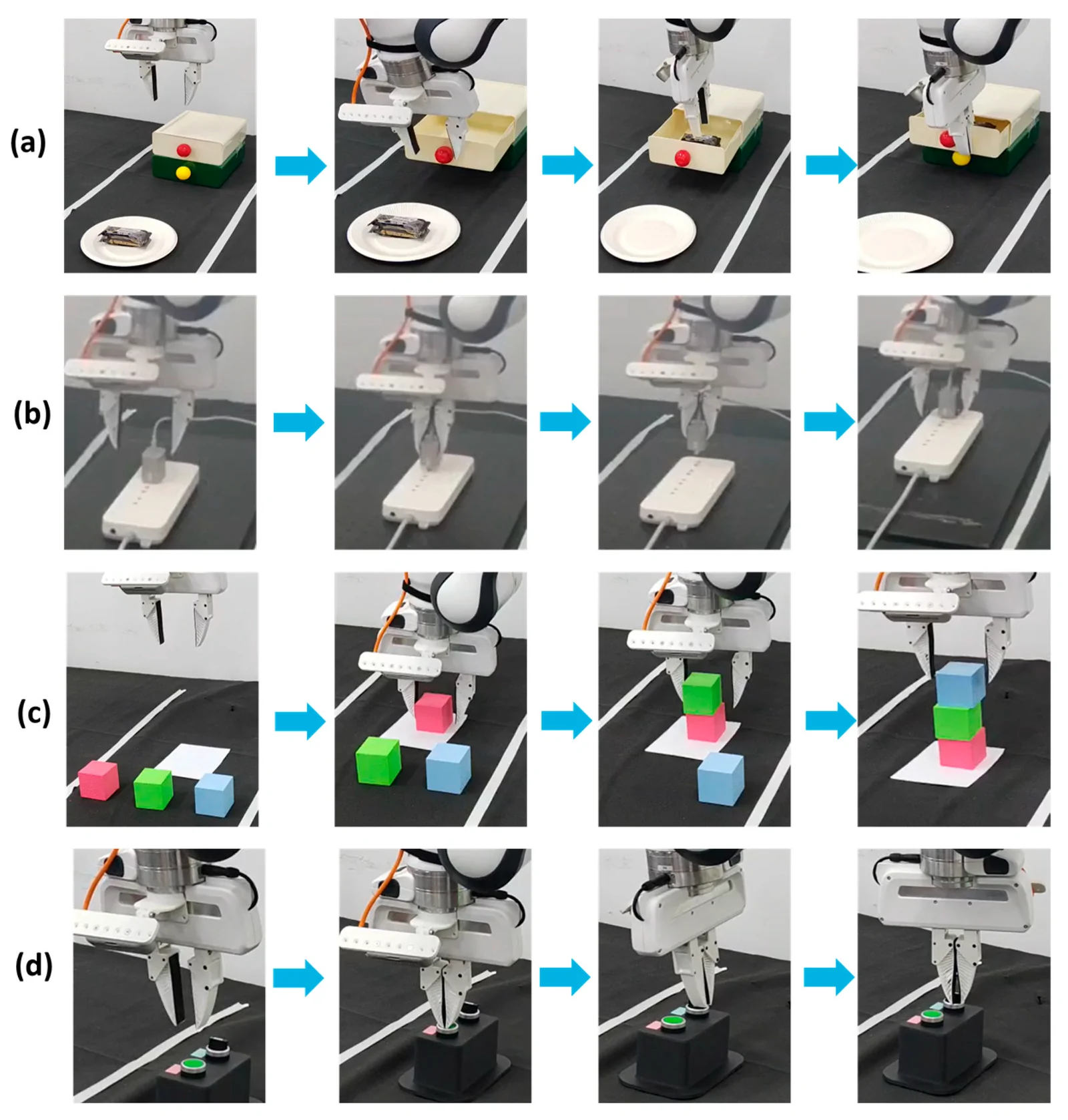
Experimental tasks for long-horizon manipulation evaluation. (**a**) Sequential pick-and-place: objects are grasped and placed in a target sequence; (**b**) Contact-rich insertion/removal under occlusion: a peg is inserted into and removed from a socket with partial visual obstruction; (**c**) Multi-object rearrangement with spatial variability: multiple objects are repositioned with varying initial and target configurations; (**d**) Ambiguous-state manipulation with symmetric configurations: objects with rotational symmetry are manipulated under perceptually ambiguous orientations.

To evaluate the contribution of multimodal cues to semantic phase perception, we conduct ablation experiments on long-horizon manipulation tasks characterized by smooth transitions and visual ambiguity. Four representative real-world tasks are selected to assess robustness as illustrated in Figure 4:

Task 1 involves sequential pick-and-place operations (open –> place –> close), requiring modeling of multi-stage dependencies.

Task 2 is a contact-rich charger insertion/removal task with severe visual occlusion, where key transition events are primarily reflected in force–torque signals rather than visual appearance.

Task 3 consists of multi-block stacking under randomized initial configurations, testing robustness to geometric variability and accumulated temporal drift.

Task 4 involves knob rotation and self-resetting switch manipulation, where identical initial and terminal states introduce ambiguity in purely visual segmentation.

For each task, 20 multimodal demonstrations were collected and manually annotated to provide ground-truth temporal boundaries. We compare three input configurations for phase segmentation: (i) Vision only, which uses multi-view RGB features. (ii) Vision + Proprioception, which additionally incorporates robot joint states and gripper states. (iii) Vision + Proprioception + Torque, which further includes external joint torque signals as physical interaction cues.

Phase-boundary F1: Phase-boundary localization is evaluated using a tolerance-based boundary F1 metric rather than the segmental temporal-IoU F1 metric. Predicted and groundtruth segmentations are first converted into frame-wise phase-label sequences. A boundary is extracted at frame t when the phase label changes between frames t−1 and t. A predicted boundary $b_{i}^{\mathrm{pred}\;}$ is counted as a true positive if it can be matched one-to-one with an unmatched ground-truth boundary $b_{j}^{\mathrm{gt}}$ satisfying

(19) $$\left|b_{i}^{\mathrm{pred}}-b_{j}^{\mathrm{gt}}\right|\leq \delta .$$

Unmatched predicted boundaries are counted as false positives, while unmatched ground-truth boundaries are counted as false negatives. Boundary precision, recall, and F1 are then computed as

(20) $$\begin{array}{c} P_{B}=\frac{N_{\mathrm{TP}}}{N_{\mathrm{TP}}+N_{\mathrm{FP}}},\;R_{B}=\frac{N_{\mathrm{TP}}}{N_{\mathrm{TP}}+N_{\mathrm{FN}}},\\ F1_{B}=\frac{2P_{B}R_{B}}{P_{B}+R_{B}}. \end{array}$$

We report δ=25 and 50 frames, corresponding to approximately 0.83 and 1.67 s at 30 Hz. A larger tolerance is less restrictive and may therefore produce a higher Boundary-F1 score.

**Figure 5:**
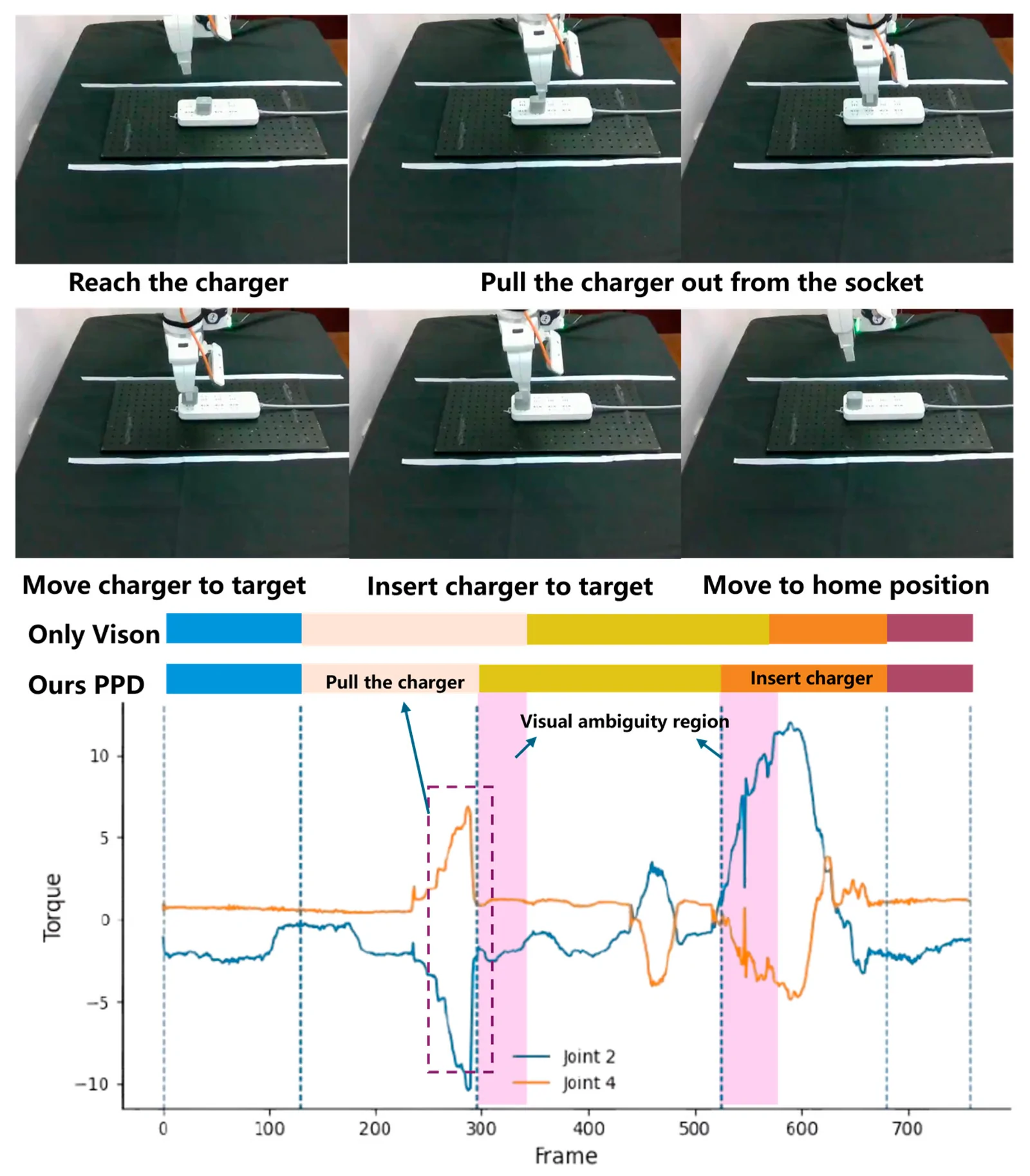
Qualitative phase segmentation in the contact-rich charger insertion/removal task. Representative keyframes show the principal task phases. The colored bars compare vision-only segmentation with the proposed Multimodal Phase-and-Primitive Detector (PPD). External joint-torque trajectories from joints J2 and J4 provide physical anchors for contact transitions. Dashed lines denote the detected phase boundaries, while the shaded regions indicate visually ambiguous contact intervals.

**Figure 6:**
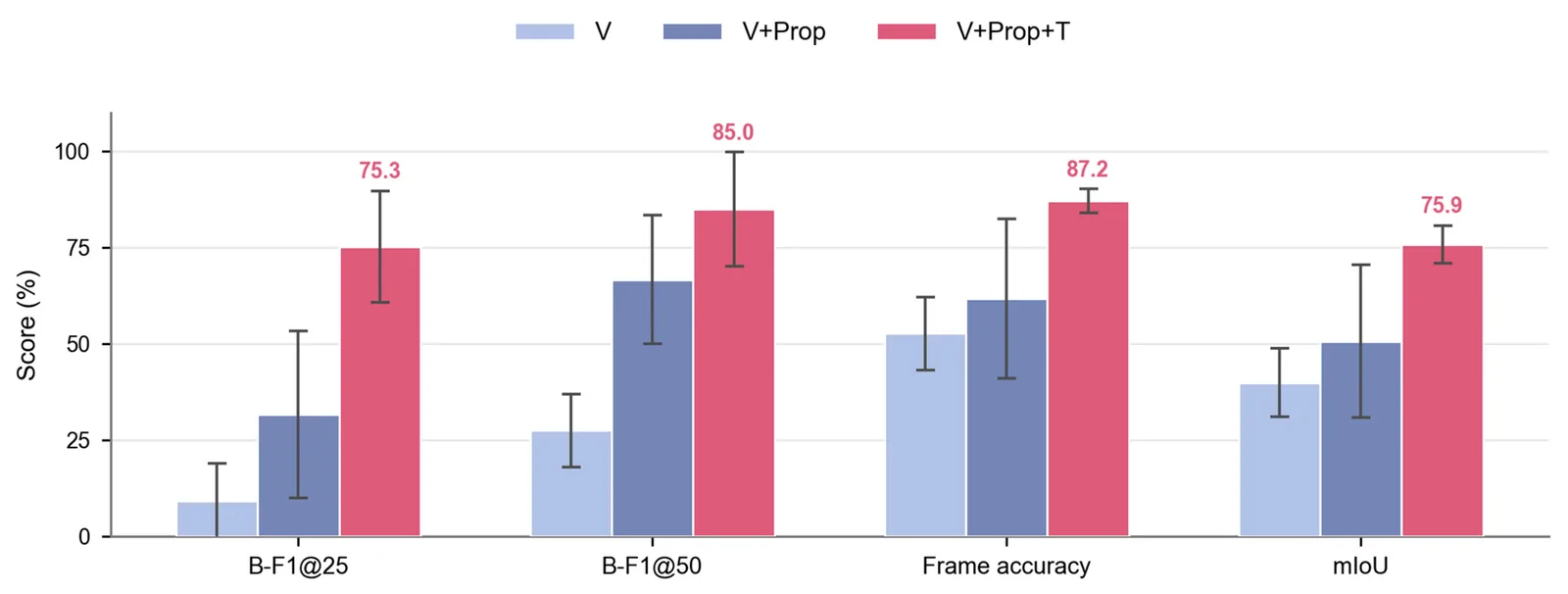
Quantitative phase-segmentation performance under different modality configurations. V denotes vision-only input, V + Prop denotes vision with robot proprioception, and V + Prop + T additionally includes external joint torque. Performance is evaluated using tolerance-based boundary F1 scores at tolerances of ±25 and ±50 frames, frame-wise accuracy, and mean intersection over union (mIoU). Error bars indicate standard deviations across the four evaluated tasks.

Figure 5, Figure 6 and Table 5 evaluate temporal phase-segmentation performance rather than robot-control success. All three modality configurations are evaluated offline on the same recorded demonstration trajectories and against the same manually annotated reference boundaries. The robot motions, inverse-kinematics solutions, low-level controller, and physical execution data are therefore identical across the three configurations; only the observation channels supplied to the segmentation pipeline are changed. Under this controlled comparison, V + Prop + T achieves the highest observed Boundary-F1, frame-wise accuracy, and mIoU values among the evaluated configurations. These results indicate that proprioceptive and external joint-torque signals provide complementary temporal cues for phase-boundary localization in the evaluated demonstrations. They should not be interpreted as evidence that force feedback is uniquely necessary for successful robot control or that a conventional controller without force feedback must fail.

### 4.3. Phase-Aware Execution and Failure Recovery

To evaluate phase-aware graph reasoning under long-horizon disturbances, we compare structured execution with monolithic and sequential baselines. All skill-based methods share identical node-level ACT policies, whereas the monolithic ACT baseline is trained on the same demonstrations using a comparable backbone capacity.

Using the baselines and fairness protocol defined in Section 4.1, we evaluate whether explicit phase-aware graph execution improves robustness under long-horizon disturbances. The comparison includes monolithic ACT, Flat Primitive Sequence, the SayCan-inspired planner, and the proposed MPSG-Control. All skill-based methods share identical node-level ACT policies.

Evaluation is conducted under two conditions: D1 denotes nominal execution without external intervention. D2 introduces runtime disturbances, including object displacement, externally completed subtasks, state reset after partial execution, and visual occlusion. These disturbances are designed to test whether the policy blindly advances according to a fixed temporal order or adapts according to perceived phase feasibility. External subtask completion denotes a condition in which an experimenter physically completes an upcoming subtask before the corresponding policy is executed, such as opening an already scheduled drawer or placing an object at its intended intermediate location. This disturbance tests whether the executor can recognize that the nominal phase has already been satisfied and skip the redundant phase. Partial-execution state reset denotes a condition in which, after one or more phases have been completed, the environment is returned to a valid state associated with an earlier phase. This disturbance tests whether the executor can redirect the graph pointer and resume from a physically consistent phase rather than continuing the original temporal sequence. Object displacement denotes a controlled change in the position of the task-relevant object during execution. Visual occlusion denotes temporary blocking of one or more task-relevant visual observations during a phase-transition interval. Grasp failures were not manually injected as a predefined disturbance category but could occur naturally during autonomous execution. Such failures could trigger the recovery mechanism of methods supporting rollback. Because recovery opportunities were not prospectively logged as an independent event-level variable with a complete denominator, we do not report a standalone recovery-success rate. Instead, the effect of recovery is evaluated through task-level success and premature-transition behavior under D2, together with the rollback ablation in Section 4.3.

For each task and each evaluated method, 20 independent execution trials were conducted under the nominal condition D1 and another 20 trials were conducted under the disturbed condition D2. Thus, each method was evaluated in 40 trials per task. Trials were reset independently, and no execution trajectory was reused across repetitions. At the beginning of each trial, the environment, graph pointer, recovery counter, and temporary graph constraints were reset to their predefined initial states. The frozen MPSG, perceptual anchors, and policy checkpoints were identical across repetitions; therefore, no trial could influence any subsequent trial. For each task, the 20 D2 trials followed a predefined disturbance composition: eight trials involved external completion of a subtask, eight trials involved resetting the environment to a previously encountered state after partial execution, two trials involved displacement of the manipulated object, and two trials involved temporary visual occlusion. The order of these disturbance instances was randomized across trials. The same disturbance composition and initialization protocol were used for all evaluated methods.

**Figure 7:**
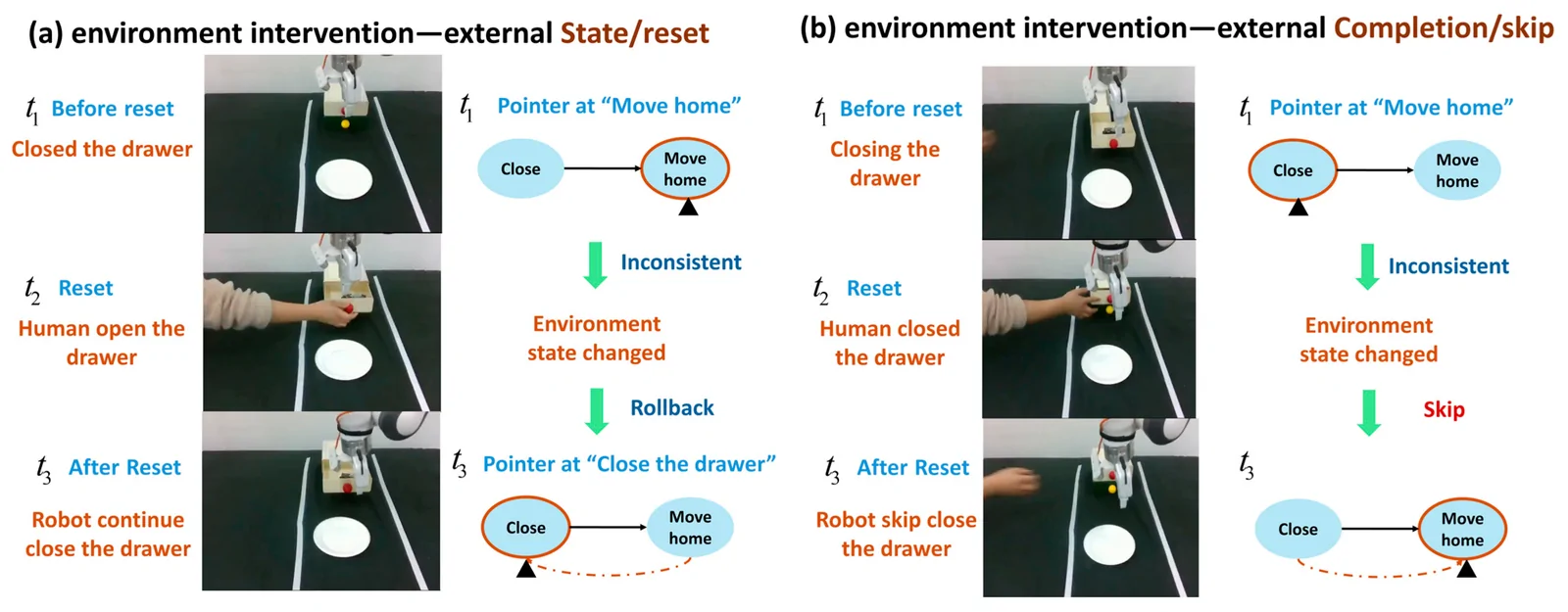
Perception-grounded graph reconfiguration under structured runtime disturbances. (**a**) After the environment is reset to an earlier valid state, MPSG detects the phase–state inconsistency and rolls the graph pointer back from Move Home to Close. (**b**) When the Close subtask is completed externally, MPSG recognizes that the corresponding phase is already satisfied and skips the redundant node. The node-level skill policies remain unchanged in both cases. The triangle indicates the current active node, and dashed lines represent robust execution paths.

As shown in Figure 7, MPSG enables structured responses to environmental inconsistencies by reassigning execution pointers under state resets and suppressing redundant phases when subtasks are externally completed. These adjustments are achieved through graph-level reasoning without modifying low-level policies.

A trial was considered successful when all required subtasks were completed, the final environment state satisfied the task-specific success criteria, and no manual intervention, safety termination, or execution timeout occurred. The task success rate is defined as (TSR)

(21) $$\mathrm{TSR}=\frac{N_{\mathrm{succ}}}{N_{\mathrm{trial}}},$$

where $N_{\mathrm{succ}}$ and $N_{\mathrm{trial}}$ denote the numbers of successful and total execution trials, respectively.

A premature transition was recorded when the executor activated the next primitive before the task-specific completion condition of the current primitive had been satisfied. Under D2, the trial-level premature transition rate is defined as

(22) $${\mathrm{PTR}}_{D2}=\frac{N_{\mathrm{trial}}^{\mathrm{PT}}}{N_{\mathrm{trial}}^{D2}},$$

where $N_{\mathrm{trial}}^{\mathrm{PT}}$ is the number of D2 trials containing at least one premature transition and $N_{\mathrm{trial}}^{D2}=20$. Multiple premature transitions within the same trial are counted only once. Unlike frame-wise segmentation accuracy, PTR is an online execution-level metric. Under D2, the physical environment is modified after robot execution has begun, and the executor must retain or update the graph pointer according to the newly observed phase feasibility. A premature transition indicates that the execution structure failed to respond before an inconsistent successor primitive was activated. Therefore, a lower PTR reflects a stronger ability to suppress state-inconsistent phase progression under runtime disturbances. PTR does not measure the wall-clock delay of a successful graph update. Instead, it evaluates whether the online response occurs before an incorrect phase transition is executed. The online MPSG traversal and node-level policy operate at 30 Hz, while the low-level robot controller operates at 1 kHz.

As reported in Table 6, MPSG consistently outperforms baselines, achieving higher success rates and reduced PTR under D2 disturbances. Task 4 introduces a state-symmetric manipulation setting where visually indistinguishable configurations lead to ambiguity in phase identification. Such ambiguity causes both ACT and Flat Primitive Sequence policies to suffer from incorrect phase progression due to the absence of explicit structural disambiguation. MPSG mitigates this issue through phase-level consistency tracking and graph-constrained execution, resulting in improved robustness under state aliasing conditions.

PTR is not reported for Task4 because its interaction structure does not provide a unique ordered successor relation for defining premature activation. Therefore, the trial-level PTR analysis is restricted to Tasks 1–3, whereas TSR is reported for all four tasks. For Task1, Flat Primitive Sequence exhibits a trial-level PTR of 100% under D2, meaning that every disturbed trial contains at least one activation of a successor primitive before the completion condition of the current primitive is satisfied. Nevertheless, its D2 TSR remains 35% (7/20), because seven trials eventually reach the final task goal despite the premature transition. This result shows that a premature transition does not necessarily cause immediate terminal failure: in some cases, the downstream low-level policy can tolerate a moderate phase–state mismatch. However, consistently premature progression reduces execution reliability. TSR and PTR therefore characterize different aspects of execution: TSR measures the terminal task outcome, whereas PTR measures the correctness of phase progression under runtime disturbances.

To isolate the structural contributions of MPSG, we conduct an ablation study under the disturbed condition D2. No language instruction is issued during this experiment, and PPC is excluded from the ablation factors. All variants use identical node-level ACT policies and differ only in their high-level execution structures. We compare four variants: (1) MPSG (Full), which retains perceptual gating, graph-based forward transitions, skipping, and rollback; (2) *w*/*o* Rollback, which removes backward recovery transitions while retaining perception-conditioned node selection and forward skipping; (3) *w*/*o* Perceptual Gating, which retains the graph topology and rollback edges but removes observation-conditioned feasibility assessment; and (4) Flat Primitive Sequence, which executes the primitive policies in a fixed order without graph-based selection, skipping, or rollback.

As shown in Table 7, removing rollback substantially reduces TSR because local failures can no longer be redirected to a feasible predecessor phase. Removing perceptual gating markedly increases PTR, indicating that graph connectivity alone is insufficient when candidate nodes are not verified against the current multimodal state. Flat Primitive Sequence produces the weakest disturbance adaptation because it lacks both state-conditioned node selection and recovery transitions. These results show that perceptual gating and rollback provide complementary functions: the former prevents infeasible forward progression, whereas the latter supports localized recovery after execution inconsistencies.

### 4.4. Structural Controllability via Hard Topological Constraints

PPC is evaluated separately from the MPSG structural ablation because it serves a different functional role. Perceptual gating and rollback determine whether the execution graph can progress and recover consistently under physical disturbances, whereas PPC converts language instructions into graph-level ordering constraints. Accordingly, PPC is evaluated using prompt-conditioned order compliance and task success. Recovery and rollback robustness are evaluated separately in Section 4.3 through disturbed-condition execution and structural ablation.

To evaluate whether language instructions can reliably induce execution-time reconfiguration, we study Promptable Phase Control (PPC) as a mechanism for mapping semantic prompts into graph-level constraints over the MPSG execution structure. Unlike approaches that treat language as a soft conditioning signal, PPC operates directly on the phase graph by applying deterministic topological masks. This enables controllable modification of execution order and activation constraints without the need to retrain low-level policies. To isolate the impact of the execution structure from control capability, we compare PPC against two baselines sharing identical low-level skill primitives:

**LC-ACT:** A stage-conditioned ACT baseline inspired by the stage-conditioning formulation of StageACT. The prompt is mapped to the existing semantic primitive vocabulary, and the currently requested primitive is encoded as a one-hot stage vector. This vector is projected to the ACT hidden dimension and introduced as an additional Transformer conditioning token. All other ACT components and training hyperparameters are unchanged.

Flat Planner: The instruction is parsed once into a fixed primitive sequence before execution. The corresponding frozen node-level ACT policies are invoked sequentially without online candidate re-scoring, perceptual graph gating, skipping, rollback, or recovery.

The robot-level PPC evaluation uses Open–Close–Open as a representative reordering case. This experiment is intended to evaluate whether a natural-language-specified order over existing primitives can be compiled into a valid MPSG constraint and executed without retraining the underlying policies; it is not intended to establish unrestricted natural-language or arbitrary graph-generation capability.

We evaluate prompt-driven task-order reconfiguration by specifying an execution order that was not used to train the low-level primitive policies. All requested actions are drawn from the same previously trained primitive library, and the same node-level ACT policies remain frozen throughout evaluation. Thus, the experiment evaluates whether graph-level constraints can reorganize known primitives at execution time rather than whether the system can acquire unseen motor skills. LC-ACT tends to follow its learned temporal priors and may deviate from the requested ordering under ambiguity. In contrast, PPC compiles the instruction into deterministic graph-level ordering constraints, resulting in higher order compliance while preserving the recovery edges of the underlying MPSG. These results demonstrate training-free primitive-order reconfiguration within the evaluated skill library and task domain. PPC is evaluated through prompt-driven task reordering.

**Figure 8:**
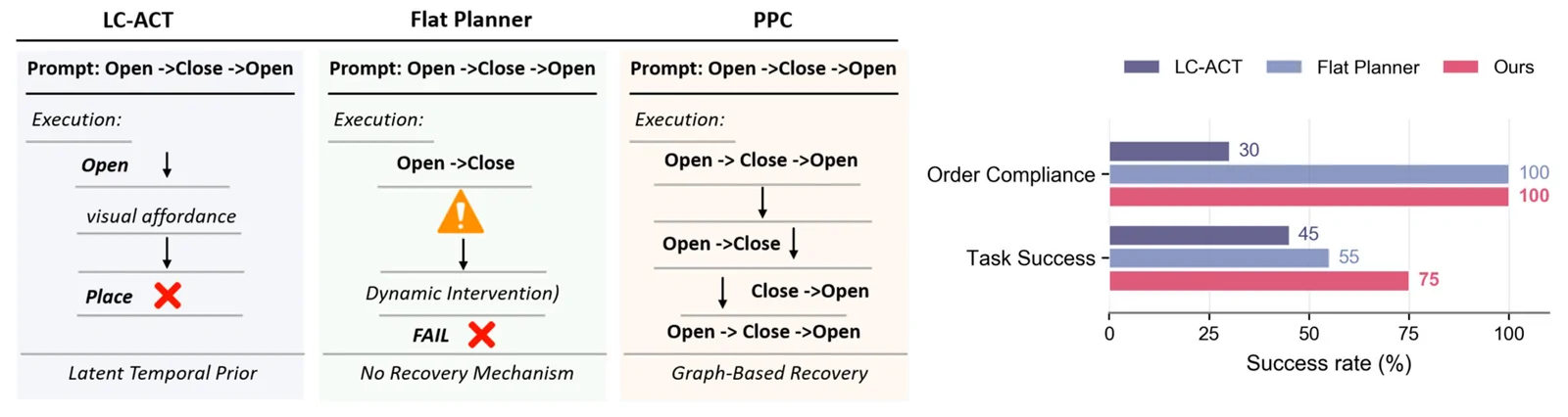
Performance comparison under prompt-driven task-order reconfiguration (Open -> Close -> Open). (**Left**) Qualitative execution flows. (**Right**) Quantitative results highlighting the trade-off between order compliance and task success across LC-ACT, Flat Planner, and the proposed PPC framework.

The quantitative results, summarized in Table 8, validate the structural advantages of our approach. LC-ACT achieves only 30% (6/20) order compliance and 45% (9/20) task success, indicating that semantic conditioning alone does not reliably enforce the requested execution order. Flat Planner achieves perfect order compliance (20/20) but only 55% (11/20) task success, showing that explicitly specifying the desired primitive order does not by itself ensure robust task execution. MPSG-Control achieves both 100% (20/20) order compliance and the highest observed task-success rate of 75% (15/20), supporting the benefit of combining hard ordering constraints with perception-grounded graph execution in the evaluated reordering case. These results indicate that separating semantic instruction parsing from perception-grounded graph execution can preserve strict order control while improving disturbance recovery in the tested setting. This experiment is intended to evaluate execution-time reconfiguration of previously learned primitives rather than the general language-understanding capability of the API model. Perception-triggered skipping, pointer reassignment, and rollback-based recovery are evaluated separately in Figure 8.

## 5. Discussion

The experimental results support the central hypothesis that explicit phase-level organization can improve the robustness and controllability of long-horizon robotic manipulation. In contrast to monolithic visuomotor policies, which implicitly couple perception, task progression, and motor execution within a single representation, the proposed framework introduces an intermediate phase abstraction between low-level skill execution and high-level task control. This abstraction allows local motor behavior to remain stable within each phase, while the global execution structure can be adapted through perception-grounded phase transitions.

In the evaluated contact-rich tasks, vision-only observations provided insufficient temporal cues for reliable phase-boundary localization. In contact-rich or partially occluded manipulation, critical phase transitions are often reflected in proprioceptive and force–torque variations rather than in visually salient image changes. By incorporating physical interaction cues into phase boundary detection, PPD produces more temporally consistent phase segments and reduces visually induced over-segmentation. This supports the biological motivation of the proposed framework, in which phase transitions are driven by sensory feedback rather than by a fixed temporal schedule.

The MPSG further demonstrates that graph-structured execution provides advantages over fixed sequential skill execution. A flat sequence can only advance according to a predefined order, making it vulnerable when a subtask fails, is externally completed, or becomes temporarily infeasible. In contrast, MPSG evaluates phase feasibility through multimodal perceptual anchors and transition priors. This enables the robot to suppress premature transitions, skip redundant phases, or roll back to a recoverable predecessor when the current phase becomes inconsistent with the observed state. As a result, local disturbances are handled at the phase level rather than propagating through the entire long-horizon task.

Promptable Phase Control (PPC) provides a complementary mechanism for high-level controllability. Its role is not to improve low-level motor execution directly, but to expose the phase graph as a structured interface for semantic intervention. In this paper, PPC is evaluated through prompt-driven task reordering, where language instructions specify a desired execution order that differs from the nominal sequence. By compiling such instructions into deterministic graph-level ordering constraints, PPC achieves strict order compliance while keeping the underlying low-level skill policies unchanged. Within the evaluated Open–Close–Open setting, representing the language instruction as a hard graph-level ordering constraint produced higher observed order compliance than the evaluated soft-conditioning baseline.

Scalability is primarily determined by the local branching structure of MPSG rather than by the total number of graph nodes. At each graph decision, the executor evaluates only the nominal, skipping, and validated recovery candidates connected to the active node, instead of performing a global search over the complete graph. For a fixed-dimensional perceptual representation, the resulting online traversal cost is linear in the number of local candidates. Nevertheless, graph storage, context-specific node duplication, and node-level policy storage increase as the task library expands. A detailed analysis of traversal complexity, graph expansion, memory growth, and primitive transferability is provided in Appendix C. Several limitations remain. First, the current graph topology depends on phase segments and recovery traces observed in demonstrations or validated during execution; therefore, recovery behaviors outside the collected experience may not be available unless they are discovered through additional interaction. Second, the primitive vocabulary remains predefined, which may limit scalability to more open-ended manipulation scenarios. Third, although PPC uses a constrained graph-edit grammar, the present experiments focus on task-order reconfiguration rather than more complex operations such as new skill insertion or open-ended subgoal generation. Finally, the current graph traversal relies on relatively simple perceptual matching and transition priors; more expressive uncertainty modeling may further improve robustness under severe ambiguity or partial observability.

Future work will explore automatic primitive discovery, uncertainty-aware graph traversal, and broader forms of promptable graph editing beyond task reordering. Another promising direction is to combine phase-aware graph execution with compliant low-level control, so that structural recovery at the task level and physical adaptation at the contact level can be jointly optimized for more complex real-world manipulation tasks.

## 6. Conclusions

This paper presented MPSG-Control, a functionally bio-inspired, phase-aware framework for structured execution in multi-stage robotic manipulation. The framework represents a demonstrated task as transitions among perception-grounded motor phases rather than as a single monolithic visuomotor trajectory. The Multimodal Phase-and-Primitive Detector uses visual, proprioceptive, and force–torque observations to identify phase segments; the Multimodal Perception Skill Graph organizes the resulting segments into node-level skill policies with nominal, skipping, rollback, and recovery transitions; and Promptable Phase Control converts a language instruction into an ordering constraint over previously trained graph nodes. Within the four evaluated manipulation tasks, the tested robot platform, and the predefined disturbance protocol, the experimental results indicate that multimodal phase perception can improve phase-segmentation accuracy relative to the evaluated reduced-modality variants. Under the tested runtime disturbances, perception-gated graph execution produced fewer premature phase transitions and higher observed task-success rates than the evaluated monolithic and fixed-sequence baselines. The prompt-driven experiment further showed that the existing Open and Close primitives could be executed in the requested open –> close –> open order without retraining their node-level policies. These results support the usefulness of explicit phase-level organization for the evaluated tasks, but they should not be interpreted as evidence of general robustness across arbitrary long-horizon manipulation problems. Likewise, the language experiment demonstrates task-order reconfiguration over an existing primitive library rather than open-ended language understanding, unseen-skill acquisition, or generalization to new objects, task structures, and environments. *See Supplementary Materials for additional experimental videos.*

Future work will evaluate the framework on larger task families, more diverse objects and environments, and broader prompt variations. Additional directions include automatic primitive discovery, uncertainty-aware graph traversal, scalable graph maintenance, and more general graph operations beyond task-order reconfiguration.

## Figures and Tables

**Table 1:** Positioning of the proposed framework against representative related approaches. ‘Yes’ denotes explicit support, ‘Partial’; denotes partial or indirect support, and ‘No’; denotes that the capability is not explicitly supported. The novelty of the present work lies not in proposing a new CLIP [31], MS-TCN, or ACT backbone in isolation, but in integrating multimodal phase abstraction, perception-grounded graph execution, rollback-based recovery, and language-to-graph hard constraints into a unified long-horizon manipulation framework.

Representative Approach	Explicit Phase Abstraction	Multimodal Physical Grounding	Runtime Reconfiguration	Rollback/Recovery	Language as Hard Structural Constraint	Main Limitation Relative to This Work
Monolithic visuomotor policies (e.g., ACT [9], RT-1 [11], Diffusion Policy [12])	No	No	No	No	No	Strong low-level control, but task progression remains implicit and execution is brittle under disturbances.
Temporal segmentation methods (e.g., MS-TCN/MS-TCN++ [32,33])	Yes	No	No	No	No	Provide temporal segmentation only; they do not define an execution structure for online manipulation control.
Hierarchical/skill- decomposition methods (e.g., options, latent plans, hierarchical predictors [18,21,22])	Partial	No	Partial	No	No	Improve compositionality, but typically rely on predefined boundaries or fixed execution hierarchies without explicit recovery structure.
Skill-graph/skill-chaining methods (e.g., generative skill chaining [23])	Partial	No	Partial	Partial	Partial	Capture reusable task structure, but usually operate on static or planning-time graphs without perception-grounded phase gating.
Language-conditioned planning/control (e.g., zero-shot planners, SayCan-style methods, RT-2, OpenVLA [13,15,29,30])	No	No	Partial	No	No	Language influences execution, but typically as a soft planner/policy condition rather than a deterministic graph-level structural constraint.
Proposed MPSG-Control	Yes	Yes	Yes	Yes	Yes	Unifies multimodal phase abstraction, perception-grounded graph execution, rollback-based recovery, and promptable graph-level reconfiguration in one framework.

**Table 2:** Mapping between biological motor-control principles and their computational abstractions in the proposed framework. The framework abstracts functional organization principles rather than reproducing detailed neural circuitry or physiological dynamics.

Biological Principle	Functional Interpretation	Computational Abstraction	Framework Component	Corresponding Experimental Hypothesis
Hierarchical motor organization	High-level task intention is separated from lower- level motor execution.	Task-level graph constraints are separated from frozen node-level visuomotor policies.	PPC and MPSG	High-level task-order changes can be imposed without retraining low-level skill policies.
Reusable motor primitives	Complex behavior is composed from locally stable and reusable motor units.	Each graph node encapsulates a node-specific ACT policy and its multimodal perceptual anchor.	MPSG nodes	Reusable primitives can be recombined across different phase sequences.
Sensory-feedback- dependent phase transition	Behavioral transitions are triggered by interaction evidence rather than elapsed time alone.	Visual, proprioceptive, and force–torque cues determine phase boundaries and transition feasibility.	PPD and perceptual gating	Multimodal physical cues improve boundary detection and reduce premature transitions under ambiguity.
Localized corrective response	Disturbances are corrected locally to limit error propagation across the complete behavior.	Skipping, rollback, and recovery edges redirect execution to a feasible phase-level predecessor or successor.	MPSG recovery logic	Phase-level recovery improves task success under runtime disturbances and partial failures.
Goal-dependent reorganization	Previously learned motor units can be reorganized according to changing task intention.	Language instructions are compiled into deterministic graph-level ordering and activation constraints.	PPC	Previously trained primitives can be reordered at execution time while remaining physically feasible.

**Table 3:** Main notation used in the proposed framework.

Symbol	Definition	Symbol	Definition	Symbol	Definition
t	Time index	r	Camera-view index	c	Primitive-class index
k	Phase-segment index	i,j	Graph-node indices	T	Number of frames in a demonstration
$O_{t}$	Multimodal observation at time t	$h_{t}$	Directed perception skill graph	$\nu_{i}$	The i-th graph node
B	Internal phase-boundary set	G	Node-specific ACT policy	$M_{t}$	Active node-mask set
$\phi_{i}$	Multimodal perceptual anchor of node $\nu_{i}$	$\pi_{i}$	Semantic label assigned to node	m	Dummy normalization index
L	Semantic primitive-label	$l_{i}$	Empirical transition prior	${\bar{P}}_{t}(j|i_{t})$	Observation-conditioned transition probability
$N^{+}(i)$	Outgoing-neighbor set of $v_{i}$	$P_{E}(j|i)$			

**Table 4:** Implementation settings and fairness controls for the evaluated methods. “Same” indicates that the corresponding configuration was shared with the proposed method.

Method	Demonstration Corpus	Observation Streams	Low-Level Policy	Policy Capacity	Robot Controller	Computer Platform
Monolithic ACT	Same	Same	ACT, full trajectory	Comparable	Same	Same
Flat Primitive Sequence	Same	Same	Same node-level ACT	Same	Same	Same
SayCan-inspired planner	Same	Same	Same node-level ACT	Same	Same	Same
LC-ACT	Same	Same + language	ACT with language condition	Matched	Same	Same
Flat Planner	Same	Same	Same node-level ACT	Same	Same	Same
Proposed MPSG-Control	Same	Same	Same node-level ACT	Same	Same	Same

**Table 5:** Quantitative phase-segmentation results under different modality configurations.

Input Configuration	Boundary-F1@ ± 25 Frames	Boundary-F1@ ± 50 Frames	Frame Acc (%)	mIoU (%)
V	9.2 ± 9.8	27.5 ± 9.5	52.7 ± 9.5	39.9 ± 8.9
V + Prop	31.7 ± 21.7	66.7 ± 16.7	61.7 ± 20.7	50.7 ± 19.8
**V + Prop + T**	**75.3 ± 14.5**	**85.0 ± 14.8**	**87.2 ± 3.1**	**75.9 ± 4.9**

**Table 6:** Long-Horizon Execution Robustness Across Structured Conditions.

Task	Method	TSR D1/D2 ↑	PTR ↓ (D2)
Task1	ACT	16/20/9/20	12/20
	Flat Primitive Sequence	16/20/7/20	20/20
	SayCan-inspired planner	18/20/10/20	9/20
	**Ours**	**18/20/16/20**	**3/20**
Task2	ACT	13/20/4/20	15/20
	Flat Primitive Sequence	11/20/6/20	20/20
	SayCan-inspired planner	12/20/7/20	11/20
	**Ours**	**16/20/15/20**	**4/20**
Task3	ACT	12/20/7/20	11/20
	Flat Primitive Sequence	13/20/4/20	20/20
	SayCan-inspired planner	12/20/7/20	11/20
	**Ours**	**16/20/16/20**	**1/20**
Task4	ACT	4/20/0/20	N/A
	Flat Primitive Sequence	6/20/0/20	N/A
	SayCan-inspired planner	6/20/0/20	N/A
	**Ours**	**15/20/12/20**	**N/A**

**Table 7:** Structural ablation under D2. PTR is averaged over Tasks 1–3, for which premature-transition events are explicitly defined, whereas TSR is averaged over Tasks 1–4.

Variant	TSR	PTR
MPSG (Full)	73.75%	13.3%
*w*/*o* Rollback	40%	70%
*w*/*o* Perceptual Gating	55%	65%
Flat Primitive Sequence	21.25%	100%

**Table 8:** Quantitative Comparison of Execution Controllability and Robustness.

Method	Order Compliance	Task Success
LC-ACT	6/20 (30%)	9/20 (45%)
Flat Planner	20/20 (100%)	11/20 (55%)
**Ours**	**20/20 (100%)**	**15/20 (75%)**

## Data Availability

The source code, trained model checkpoints, configuration files, manual phase annotations, task definitions, disturbance protocols, and evaluation scripts will be made publicly available upon publication at [repository (https://github.com/cheng9911/mpsg_control) (accessed on 11 August 2026)]. The complete raw multimodal dataset will be available from the corresponding author upon reasonable request. MPSG-Control—Bio-Inspired Phase-Aware Skill Graph (https://cheng9911.github.io/PhaseAct-Website/index.html) (accessed on 11 August 2026).

## Supplementary Materials

The following supporting information can be downloaded at https://www.mdpi.com/article/10.3390/biomimetics11090622/s1. The following videos are provided as qualitative demonstrations of the proposed framework. Each video shows a representative execution example and is intended to illustrate the corresponding graph-transition or execution mechanism. The videos are not used to compute or independently validate the aggregate quantitative results reported in Section 4. Video S1: Long-horizon robustness under runtime disturbances (mpsg.mp4). This video presents representative long-horizon executions of MPSG-Control under manually introduced runtime disturbances. The demonstrated cases illustrate how the executor responds to changes in the physical task state through perception-grounded graph adaptation, including phase retention, pointer reassignment, skipping, and rollback when applicable; Video S2: Nominal execution of Task 1: Drawer manipulation. This video shows successful nominal execution of the complete drawer-manipulation sequence, including drawer opening, cookie grasping, transfer and placement into the drawer, drawer closing, and return to the initial configuration. No external disturbance is introduced. The video illustrates normal MPSG node transitions and nominal task completion; Video S3: Nominal execution of Task 2: Charger manipulation. This video shows successful nominal execution of the charger task, including approach, charger removal, transfer to the target location, insertion, release, and return. No external disturbance is introduced. The video illustrates normal graph progression through contact-rich manipulation phases; Video S4: Nominal execution of Task 3: Three-block stacking. This video shows successful nominal stacking of three blocks in the predefined sequence. Each block undergoes approach, grasp, transfer, and placement. No external disturbance is introduced. The video illustrates repeated traversal of phase-level manipulation primitives during a long-horizon task; Video S5: Nominal execution of Task 4: Button and rotary-switch manipulation. This video shows successful nominal execution of the button/rotary-switch task. No external disturbance is introduced. The video illustrates MPSG phase transitions in a manipulation sequence containing discrete interaction-state changes; Video S6: Recovery by graph-pointer reassignment (recover). This drawer-manipulation example corresponds to Figure 8 (left). After the robot has progressed to a later stage of the task, the environment is manually returned to an earlier incomplete state. MPSG detects that the current physical state is inconsistent with the active graph phase and redirects the graph pointer to a feasible predecessor phase, after which execution resumes from the restored state; Video S7: Skipping an externally completed subtask (skip). This drawer-manipulation example corresponds to Figure 8 (right). Before the corresponding robot primitive is executed, the experimenter manually completes the required subtask. MPSG recognizes that the associated phase has already been satisfied and skips the redundant primitive, allowing execution to continue from the next physically consistent graph node. These supplementary videos are selected representative trials and are provided solely to visualize the behaviors described in the manuscript. They should not be interpreted as additional experimental repetitions or as validation of the aggregate success rates. All quantitative results are derived from the independent evaluation trials reported in Section 4.

## Abbreviations

PPD	Multimodal Phase-and-Primitive Detector
MPSG	Multimodal Perception Skill Graph
PPC	Promptable Phase Control
ACT	Action Chunking Transformer
LC-ACT	Language-Conditioned Action Chunking Transformer
DoF	Degrees of Freedom
CLIP	Contrastive Language–Image Pre-training
MS-TCN	Multi-Stage Temporal Convolutional Network
TSR	Task Success Rate
PTR	Premature Transition Rate
mIoU	Mean Intersection over Union

## Appendix A. Implementation and Reproducibility Details

**Table A1:** Implementation details and hyperparameters of the PPD phase-segmentation pipeline. All settings were fixed using the development training/validation partitions before final evaluation.

(**a**) Feature extraction		
**Component**	**Setting**	**Value/Architecture**
Visual encoder	Backbone/pretrained weights	OpenCLIP ViT-B/32, laion2b_s34b_b79k
Visual embedding	Per camera	512 dimensions, ($L_{2}$)-normalized
Multi-view aggregation	Fusion before physical feature	Concatenation; 1024 dimensions
Physical representation	Input descriptor	(${\left[g_{t},m_{t},\Delta m_{t},a_{t},c_{t}\right]}^{⊤}$), 5 dimensions
Torque channels	Selected channels	(7)
Torque normalization	Numerical constant	($\epsilon =10^{-6}$)
Direction-consistency window	Window length	4 frames
Physical normalization	Normalization	Channel-wise z-score using training-set statistics
(**b**) Physical encoder + fusion network		
**Component**	**Setting**	**Value/Architecture**
Physical encoder	Architecture	(5→32→64)
	Activation	ReLU
	Normalization	LayerNorm
	Dropout	0.1 after each ReLU
Fusion input	Visual + physical	(1024 + 64 = 1088) dimensions
Fusion network	Architecture	(1088→512→256)
	Activation	GELU
	Dropout	0.2/0.1
PPD fused feature	Output	256 dimensions
(**c**) Change-point segmentation		
**Component**	**Setting**	**Value**
Change-point algorithm	Method	Binary Segmentation
Segment cost	Cost function	Squared Euclidean ($L_{2}$)
Penalty	Segmentation penalty	2.0
Minimum segment size	min_size	3 frames
Jump interval	jump	5 frames
Pseudo-label smoothing	Window	11 frames
Minimum retained phase duration	Post-processing	15 frames (0.5 s)
Number of phase classes	Task dependent	Task 1–4: 7/5/8 6
(**d**) MS-TCN		
**Component**	**Setting**	**Value**
MS-TCN	Number of stages	3
	Layers per stage	4
Temporal convolution	Dilations	{1,2,4,8}
	Kernel size	3
	Hidden dimension	128
	Dropout	0.3
Training window	Length/stride	64/8 frames
Loss	Training objective	Frame-wise cross entropy
Optimizer	Optimizer	AdamW
	Learning rate	($1\times 10^{-4}$)
	Weight decay	($1\times 10^{-4}$)
	Batch size	8
Training duration	Maximum epochs	50
	Early-stopping patience	5
LR scheduler	ReduceLROnPlateau	factor 0.5, patience 5

**Table A2:** Architecture and training configuration of the shared ACT policies used by the skill-based methods and the monolithic ACT baseline.

Item	Configuration	Item	Configuration
Camera views	Head, flan, and right	Image resolution	3×480×640 per view
Proprioceptive input	7 joint positions, gripper position, and 7 joint velocities	Gradient clipping/random seed	10.0/1000
Visual backbone	Shared ImageNet-pretrained ResNet-18	Visual fusion	Camera feature tokens concatenated in the Transformer encoder
Transformer dimension	512	Attention heads	8
Encoder/decoder layers	4/1	Feedforward dimension	3200
Activation/dropout	ReLU/0.1	VAE encoder layers	4
Observation steps	1	Action-chunk horizon	100
Temporal ensembling	Disabled	Optimizer	AdamW
Learning rate/weight decay	$10^{-5}$/$10^{-4}$	Batch size/training budget:	16/200,000 optimization steps

**Table A3:** Numerical thresholds and deterministic traversal and recovery parameters of MPSG. All values were fixed before final evaluation and kept unchanged across the evaluated tasks and baselines.

Parameter	Symbol	Value	Function
Perceptual-feasibility threshold	$\eta_{p}$	0.70	Rejects candidates inconsistent with the current multimodal state
Transition-confidence threshold	$\eta_{t}$	0.80	Minimum joint confidence for node activation
Phase-completion confidence threshold	$\eta_{c}$	0.85	Minimum graph-level confidence for accepting completion of a demonstration-defined semantic phase.
Forward-skipping threshold	$\eta_{s}$	0.90	Applies a stricter requirement to nonadjacent forward candidates
Maximum recovery attempts	$N_{\mathrm{rec}}^{\max}$	3	Prevents unbounded rollback cycles
Tie-breaking rule	—	Highest empirical transition occurrence	Resolves equal-confidence candidates
No-candidate behavior	—	Rollback or safe stop	No infeasible low-level policy is activated

**Table A4:** Baseline implementation details $S_{\mathrm{perc}}$ denotes the same multimodal perceptual compatibility score used by MPSG-Control. All ACT-based methods use the architecture and optimization settings reported in Table A2. No baseline receives additional robot demonstrations.

Method	High-Level Input	Selection/Conditioning	Low-Level Policy	Graph/Recovery
SayCan-inspired	Natural-language instruction	$0.6S_{\mathrm{perc}}+0.4S_{\mathrm{lang}}$	Same frozen node-level ACT	No
LC-ACT	Semantic stage from instruction	One-hot stage token projected to 512D and conditioned on ACT	Matched ACT, Table A2	No
Flat Planner	Natural-language instruction	Fixed parsed primitive sequence	Same frozen node-level ACT	No
MPSG-Control	Language multimodal observation	Perception-gated graph traversal	Same frozen node-level ACT	Yes

**Table A5:** PPC language-parser configuration.

Parameter	Setting
Semantic parser	Kimi K2.6
Invocation	On demand, once per new instruction
Output format	JSON only
Number of candidates	1
Temperature	API default
top-p	API default
Maximum output tokens	API default
Execution timing	Parsed before robot execution in reported experiment
Invalid output	Rejected; current graph retained

**Table A6:** Task-specific primitive-completion and trial-success criteria used in the robot experiments.

Task	Primitive	Completion Condition	Geometric/Force Threshold	Failure Condition
Task 1: Drawer manipulation	Open drawer	Drawer reaches the demonstrated open state and the corresponding phase is recognized as complete	No manually specified displacement threshold; completion is referenced to the demonstrated open-state distribution	Failure if the intended state cannot be reached or execution is terminated
	Grasp cookie	Cookie is securely grasped and remains coupled to the gripper during subsequent motion	No fixed grasp-position threshold; gripper state and multimodal phase observation are used	Failed grasp may trigger local recovery
	Move/Place	Grasped cookie is transported into the drawer and released in the intended drawer region	No fixed Cartesian tolerance; small placement deviations are accepted if the cookie remains inside the drawer and the task can proceed	Failure if the cookie is lost or cannot be placed in the drawer
	Close drawer	Drawer returns to the demonstrated closed state	No manually specified displacement threshold	Failure if closure cannot be completed
	Return	Robot returns to the nominal initial/home configuration after task completion	Demonstration-defined terminal configuration	Failure if execution is terminated before completion
Task 2: Charger manipulation	Approach	End effector reaches the demonstrated pre-removal region of the charger	No fixed Cartesian threshold	Failure if the subsequent removal primitive cannot be initiated
	Remove	Charger is completely disengaged from its original socket	No fixed extraction-distance or force threshold; torque/contact cues support phase recognition	Failed removal may trigger recovery
	Move	Removed charger is transported to the target socket	Demonstration-defined target approach state	Failure if the object is lost or target cannot be reached
	Insert	Charger is successfully seated in the target socket and remains inserted	No manually specified insertion-depth or force threshold; successful physical engagement defines completion	Failure if insertion cannot be completed or safety termination occurs
	Release/Return	Gripper releases the charger and robot returns to the initial/home state	No additional geometric threshold	Failure if the terminal task state is not reached
Task 3: Three-block stacking	Approach	End effector reaches the demonstrated pre-grasp state of the currently scheduled block	Demonstration-defined state	Failure if grasp cannot be initiated
	Grasp	Current block is successfully grasped and remains attached during transport	No fixed grasp-pose threshold	Failed grasp may trigger recovery
	Move	Block is transported toward its designated stacking location	Demonstration-defined transport endpoint	Failure if the block is dropped or the target cannot be reached
	Place	Block is released and remains stably positioned in the required stack	No fixed Cartesian placement tolerance; stable successful stacking defines completion	Failure if the block falls or prevents continuation
	Sequence completion	The above primitives are repeated for the three blocks in the predefined order	—	Trial fails if the final three-block stack is not successfully formed
Task 4: Button/rotary-switch manipulation	Approach	End effector reaches the demonstrated interaction state associated with the selected control element	Demonstration-defined state	Failure if interaction cannot be initiated
	Press/Rotate	Button or rotary switch reaches the intended discrete functional state	No manually specified travel/angle threshold; completion is defined by the resulting device state	Failure if the requested device-state transition is not achieved
	Release/Reset	Contact is released and the required post-interaction state is observed	No additional geometric threshold	Failure if the expected terminal state is absent

**Note:** No primitive-specific timeout was used. All four tasks were subject to a common global trial timeout of $T_{\mathrm{trail}}^{\mathrm{Max}}=180\;s$. If the required final task state was not reached within this interval, the trial was terminated and recorded as unsuccessful. The failure conditions listed in the last column describe additional primitive- or task-level termination conditions. Final task success criteria: Task 1 requires drawer opening, cookie grasping and placement, drawer closure, and return; Task 2 requires charger removal, transfer, insertion, release, and return; Task 3 requires formation of the complete three-block stack; and Task 4 requires completion of the prescribed button/rotary-switch state transitions.

## Appendix B. Runtime Deployment and Computational Timing

**Table A7:** Summarizes the deployment modes and timing characteristics of the principal system components. The reported system uses an asynchronous multi-rate architecture rather than executing all modules sequentially within a single control cycle. Consequently, the individual latencies in the table should not be directly summed to obtain the 1 kHz robot-control period.

Component	Execution Mode	Platform	Time/Frequency
Node-level ACT policy inference	Online	Jetson AGX Orin	30 Hz
Demonstration segmentation and MS-TCN refinement	Offline	Jetson AGX Orin	Before deployment
Low-level robot control	Online	Intel NUC	1 kHz
PPC language parsing	On demand	External API	1 s
MPSG graph-pointer and constraint update	On demand	Jetson AGX Orin	10 ms
Asynchronous online phase-recognition update	Online	Jetson AGX Orin	60 ms

## Appendix C. Scalability, Computational Complexity, and Transferability of MPSG

This appendix analyzes the computational and storage complexity of MPSG and clarifies the scope of the transferability claims. Let $G=\left(V,E\right)$ denote the directed skill graph, where N=|V| and M=|E| are the numbers of graph nodes and edges, respectively. For the currently active node $v_{i}$, let $d_{i}$ denote the number of enabled nominal and skipping successors, and let $r_{i}$ denote the number of validated rollback or recovery candidates. The dimensionality of each multimodal perceptual anchor is denoted by D.

### Appendix C.1. Online Graph-Traversal Complexity

MPSG does not perform a global graph search at every policy step. Given the active node $v_{i}$, the executor retrieves only its locally connected nominal, skipping, and recovery candidates. Candidate retrieval therefore requires $O\left(d_{i}+r_{i}\right)$ operations when the graph is represented using adjacency lists.

For each candidate, the executor computes a multimodal perceptual matching score, combines this score with the corresponding edge-transition prior, checks the feasibility conditions, and identifies the candidate with the largest valid transition confidence. Computing a D-dimensional perceptual similarity for all local candidates requires

$$\;O\left(\left(d_{i}+r_{i}\right)D\right)$$

time. Candidate masking, score normalization, and maximum-confidence selection additionally require $O\left(d_{i}+r_{i}\right)$ time. The overall graph decision complexity is therefore

$$\;O\left(\left(d_{i}+r_{i}\right)D\right)$$

Because D is fixed during deployment, the practical decision cost is linear in the local candidate count:

$$\;O\left(d_{i}+r_{i}\right)$$

This local-search formulation means that increasing the total number of nodes does not directly increase the cost of every graph decision, provided that the local node degree remains bounded. In the worst case of a densely connected graph, $d_{i}+r_{i}$ may grow proportionally to N, resulting in an upper-bound complexity of $O\left(ND\right)$ per graph decision.

### Appendix C.2. Graph-Expansion Complexity

Graph expansion may involve adding a validated transition edge or inserting a new context-specific node. With an adjacency-list representation, adding an edge requires constant structural insertion time once the source and target nodes have been identified. Any additional feasibility validation or duplicate-edge checking depends on the implementation and may require searching the corresponding local neighborhood.

Adding a new graph node requires storing its multimodal perceptual anchor and graph metadata, resulting in $O\left(D\right)$ additional graph-level storage. If the new node is associated with k incoming and outgoing edges, its structural insertion requires $O\left(k\right)$ additional edge-storage operations. When an independent node-specific policy with P parameters is assigned to the new node, an additional $O\left(P\right)$ model-storage cost is introduced.

The present implementation expands the graph only through validated demonstration transitions and successful recovery traces. It does not perform unconstrained online creation of arbitrary new skills or graph nodes.

### Appendix C.3. Memory Complexity

Using adjacency lists, the graph topology requires $O\left(N+M\right)$ storage. The perceptual anchors require $O\left(ND\right)$ storage. Excluding the low-level policy parameters, the complete graph-level representation therefore requires memory.

$$\;O\left(ND+M\right)$$

If every graph node maintains an independent policy containing P parameters, the upper-bound storage requirement of the complete framework becomes

$$\;O\left(N\left(P+D\right)+M\right)$$

Thus, storage increases linearly with the number of explicitly retained nodes and policies and with the number of graph edges. For sparse phase-transition graphs, M generally grows approximately linearly with N. In a theoretical densely connected graph, however, M can approach $O\left(N^{2}\right)$. Context-specific duplication of semantically similar nodes and independent storage of node-level policy checkpoints may therefore become important limitations for substantially larger task libraries.

### Appendix C.4. Transferability and Scope

The current experiments use one robot platform, a predefined primitive vocabulary, and relatively small task graphs. Accordingly, the demonstrated primitive reuse refers to recombination of previously trained primitives within the evaluated robot, object, sensing, and task domain. The present results do not establish direct transfer of the perceptual anchors or node-level ACT policies to unseen robot embodiments, new object categories, or substantially different environments.

Scaling MPSG to longer or more open-ended tasks may require hierarchical graph decomposition, clustering or merging of contextually redundant nodes, graph pruning, shared or parameter-efficient primitive policies, and uncertainty-aware retrieval of candidate nodes. These extensions are identified as future work rather than capabilities demonstrated by the current experiments.

**Table A8:** Computational and storage complexity of the principal MPSG operations.

Operation	Complexity	Explanation
Local candidate retrieval	$O(d_{i}+r_{i})$	Retrieves nominal, skipping, and validated recovery candidates of the active node
Perceptual matching and node selection	$O((d_{i}+r_{i})D)$	Computes D-dimensional matching scores and selects the highest-confidence feasible node
Worst-case graph decision	O(ND)	Occurs only when the active node is connected to a number of candidates proportional to the complete graph size
Edge insertion	Structural insertion O(1)	Excludes implementation-dependent validation and duplicate checking
Node insertion	O(D+k)	Stores one perceptual anchor and k incident edges
Node insertion with independent policy	O(P+D+k)	Includes a node-specific policy containing P parameters
Graph-level storage	O(ND+M)	Includes perceptual anchors and adjacency-list graph structure
Total storage with independent policies	O(N(P+D)+M)	Upper bound when every node stores a separate policy

## Appendix D. Wilson Confidence Intervals for Execution-Level Binary Outcomes

The execution-level task success rate (TSR), premature transition rate (PTR) and order compliance are proportions estimated from binary trial outcomes. Because several evaluated conditions contain only 20 independent trials and include boundary cases such as 0/20 and 20/20, two-sided 95% Wilson score confidence intervals are reported instead of normal-approximation intervals. For an observed event count k from n independent trials, let $\hat{p}=k/n$. The two-sided 95% Wilson score interval is computed as

(A1) $${\mathrm{CI}}_{95\%}=\frac{\hat{p}+\frac{z^{2}}{2n}\pm z\sqrt{\frac{\hat{p}(1-\hat{p})}{n}+\frac{z^{2}}{4n^{2}}}}{1+\frac{z^{2}}{n}},\;z=1.96.$$

All intervals are reported as percentages and rounded to one decimal place. The intervals quantify the sampling uncertainty of each observed proportion; they are not interpreted as formal pairwise significance tests.

**Table A9:** Two-sided 95% Wilson score confidence intervals for the task-wise TSR and trial-level PTR results reported in Table 6. Each condition contains 20 independent trials. PTR is not reported for Task 4 because a unique premature-successor criterion is not defined for its state-symmetric interaction structure.

Task	Method	TSR Under D1, Count (Rate [95% CI])	TSR Under D2, Count (Rate [95% CI])	PTR Under D2, Count (Rate [95% CI])
Task 1	ACT	16/20 (80.0% [58.4, 91.9])	9/20 (45.0% [25.8, 65.8])	12/20 (60.0% [38.7, 78.1])
Task 1	Flat Primitive Sequence	16/20 (80.0% [58.4, 91.9])	7/20 (35.0% [18.1, 56.7])	20/20 (100.0% [83.9, 100.0])
Task 1	SayCan-inspired Planner	18/20 (90.0% [69.9, 97.2])	10/20 (50.0% [29.9, 70.1])	9/20 (45.0% [25.8, 65.8])
Task 1	MPSG-Control	18/20 (90.0% [69.9, 97.2])	16/20 (80.0% [58.4, 91.9])	3/20 (15.0% [5.2, 36.0])
Task 2	ACT	13/20 (65.0% [43.3, 81.9])	4/20 (20.0% [8.1, 41.6])	15/20 (75.0% [53.1, 88.8])
Task 2	Flat Primitive Sequence	11/20 (55.0% [34.2, 74.2])	6/20 (30.0% [14.5, 51.9])	20/20 (100.0% [83.9, 100.0])
Task 2	SayCan-inspired Planner	12/20 (60.0% [38.7, 78.1])	7/20 (35.0% [18.1, 56.7])	11/20 (55.0% [34.2, 74.2])
Task 2	MPSG-Control	16/20 (80.0% [58.4, 91.9])	15/20 (75.0% [53.1, 88.8])	4/20 (20.0% [8.1, 41.6])
Task 3	ACT	12/20 (60.0% [38.7, 78.1])	7/20 (35.0% [18.1, 56.7])	11/20 (55.0% [34.2, 74.2])
Task 3	Flat Primitive Sequence	13/20 (65.0% [43.3, 81.9])	4/20 (20.0% [8.1, 41.6])	20/20 (100.0% [83.9, 100.0])
Task 3	SayCan-inspired Planner	12/20 (60.0% [38.7, 78.1])	7/20 (35.0% [18.1, 56.7])	11/20 (55.0% [34.2, 74.2])
Task 3	MPSG-Control	16/20 (80.0% [58.4, 91.9])	16/20 (80.0% [58.4, 91.9])	1/20 (5.0% [0.9, 23.6])
Task 4	ACT	4/20 (20.0% [8.1, 41.6])	0/20 (0.0% [0.0, 16.1])	N/A
Task 4	Flat Primitive Sequence	6/20 (30.0% [14.5, 51.9])	0/20 (0.0% [0.0, 16.1])	N/A
Task 4	SayCan-inspired Planner	6/20 (30.0% [14.5, 51.9])	0/20 (0.0% [0.0, 16.1])	N/A
Task 4	MPSG-Control	15/20 (75.0% [53.1, 88.8])	12/20 (60.0% [38.7, 78.1])	N/A

**Table A10:** Two-sided 95% Wilson score confidence intervals for the structural ablation results reported in Table 7. TSR is computed over 80 trials across Tasks 1–4, whereas PTR is computed over 60 disturbed trials across Tasks 1–3.

Variant	TSR, Count (Rate [95% CI])	PTR, Count (Rate [95% CI])
**MPSG-Control (Full)**	**59/80 (73.8% [63.2, 82.1])**	**8/60 (13.3% [6.9, 24.2])**
*w*/*o* Rollback	32/80 (40.0% [30.0, 51.0])	42/60 (70.0% [57.5, 80.1])
*w*/*o* Perceptual Gating	44/80 (55.0% [44.1, 65.4])	39/60 (65.0% [52.4, 75.8])
Flat Primitive Sequence	17/80 (21.2% [13.7, 31.4])	60/60 (100.0% [94.0, 100.0])

**Table A11:** Two-sided 95% Wilson score confidence intervals for the prompt-driven task-order reconfiguration results reported in Table 8.

Method	Order Compliance, Count (Rate [95% CI])	Task Success, Count (Rate [95% CI])
LC-ACT	6/20 (30.0% [14.5, 51.9])	9/20 (45.0% [25.8, 65.8])
Flat Planner	20/20 (100.0% [83.9, 100.0])	11/20 (55.0% [34.2, 74.2])
MPSG-Control	20/20 (100.0% [83.9, 100.0])	15/20 (75.0% [53.1, 88.8])
